# Global Transcriptional Regulators Fine-Tune the Translational and Metabolic Efficiency for Optimal Growth of Escherichia coli

**DOI:** 10.1128/mSystems.00001-21

**Published:** 2021-03-30

**Authors:** Mahesh S. Iyer, Ankita Pal, Sumana Srinivasan, Pramod R. Somvanshi, K. V. Venkatesh

**Affiliations:** a Department of Chemical Engineering, Indian Institute of Technology Bombay, Mumbai, India; FMRP-USP

**Keywords:** global transcriptional regulators, metabolic and translational efficiency, exponential growth, intracellular metabolites, proteome allocation, anaerobic fermentation

## Abstract

Global transcriptional regulators coordinate complex genetic interactions that bestow better adaptability for an organism against external and internal perturbations. These transcriptional regulators are known to control an enormous array of genes with diverse functionalities. However, regulator-driven molecular mechanisms that underpin precisely tuned translational and metabolic processes conducive for rapid exponential growth remain obscure. Here, we comprehensively reveal the fundamental role of global transcriptional regulators FNR, ArcA, and IHF in sustaining translational and metabolic efficiency under glucose fermentative conditions in Escherichia coli. By integrating high-throughput gene expression profiles and absolute intracellular metabolite concentrations, we illustrate that these regulators are crucial in maintaining nitrogen homeostasis, govern expression of otherwise unnecessary or hedging genes, and exert tight control on metabolic bottleneck steps. Furthermore, we characterize changes in expression and activity profiles of other coregulators associated with these dysregulated metabolic pathways, determining the regulatory interactions within the transcriptional regulatory network. Such systematic findings emphasize their importance in optimizing the proteome allocation toward metabolic enzymes as well as ribosomes, facilitating condition-specific phenotypic outcomes. Consequentially, we reveal that disruption of this inherent trade-off between ribosome and metabolic proteome economy due to the loss of regulators resulted in lowered growth rates. Moreover, our findings reinforce that the accumulations of intracellular metabolites in the event of proteome repartitions negatively affects the glucose uptake. Overall, by extending the three-partition proteome allocation theory concordant with multi-omics measurements, we elucidate the physiological consequences of loss of global regulators on central carbon metabolism restraining the organism to attain maximal biomass synthesis.

**IMPORTANCE** Cellular proteome allocation in response to environmental or internal perturbations governs their eventual phenotypic outcome. This entails striking an effective balance between amino acid biosynthesis by metabolic proteins and its consumption by ribosomes. However, the global transcriptional regulator-driven molecular mechanisms that underpin their coordination remains unexplored. Here, we emphasize that global transcriptional regulators, known to control expression of a myriad of genes, are fundamental for precisely tuning the translational and metabolic efficiencies that define the growth optimality. Towards this, we systematically characterized the single deletion effect of FNR, ArcA, and IHF regulators of Escherichia coli on exponential growth under anaerobic glucose fermentative conditions. Their absence disrupts the stringency of proteome allocation, which manifests as impairment in key metabolic processes and an accumulation of intracellular metabolites. Furthermore, by incorporating an extension to the empirical growth laws, we quantitatively demonstrate the general design principles underlying the existence of these regulators in E. coli.

## INTRODUCTION

Coordination of the proteomic resources with metabolic needs is fundamental for cell proliferation (1–3). This is majorly due to the huge energetic expenditure associated with protein synthesis constrained by ribosomal capacity. Hence, when a bacterial cell, such as Escherichia coli, is challenged with nutrient limitation, the organism remodels its proteome allocation majorly toward the metabolic proteome that facilitates enhanced uptake of nutrients and its metabolism, thereby reducing the share toward the ribosomes. On the contrary, under nutrient-rich conditions, proteome allocation toward ribosomes is augmented to enable faster growth with reduced synthesis of the metabolic proteome. Broadly, this proteome allocation theory can be explained by the trade-off existing between metabolic flux involved in generating the precursors and amino acids by the metabolic proteome and protein synthesis flux utilizing the amino acids by ribosomes, which results in balanced exponential growth (4). Such metabolic and translational efficiencies facilitated by the endogenous proteome of the central carbon metabolism and ribosomes entail bacterial growth to be precisely controlled by the joint effects of global physiological machinery (e.g., RNA polymerase, ribosomes, and alarmones) and transcriptional regulators (4–8). Global physiological machinery that propagates growth-dependent changes in gene expression and cellular decision making have been addressed under nutrient shifts and antibiotic stress conditions (4, 5, 9–12). However, the role of global transcriptional regulators causing promiscuous regulatory effects that enable the precise coordination of cellular resources for adaptation to a specific environment remains hitherto overlooked.

Towards this, we focused on global transcriptional regulators FNR (fumarate and nitrate reductase), ArcA (aerobic respiration control A), and IHF (integration host factor) that occupy the top-most hierarchy of transcriptional regulatory network in E. coli (13). FNR activates the synthesis of enzymes that function primarily in anaerobic respiration or fermentation as well as represses the synthesis of certain enzymes involved in aerobic respiration (14, 15). The presence of oxygen inhibits FNR activity, thereby affecting its regulatory effect (14, 15). On the other hand, ArcA is part of the two-component system (ArcB-ArcA) that negatively regulates the operons that function in the tricarboxylic acid (TCA) cycle, glyoxylate shunt, and the electron transport chain and the enzymes involved in fatty acid breakdown pathways (14, 15) under anaerobic or microaerobic conditions. Similar to that of FNR, the activity of ArcA is known to be influenced by the redox state of the quinone pools and oxygen levels that affect its phosphorylation state (15, 16). So far, genome-wide binding studies on these global anoxic regulators, FNR and ArcA, exploring their targeted effects in varied oxygenation and nutritional conditions have been well appreciated (17–20). Conventionally, a broad overview of the role of these regulators in sensing metabolic redox state and their central carbon metabolic responses have been thoroughly investigated using gene expression and metabolic flux analyses in anaerobic, microaerobic, and anaerobic-aerobic transition environments (14–26). Similarly, IHF has been well characterized as a nucleoid protein and for its regulatory effects under respiratory and diverse nutrient conditions (21–24). Genome-wide binding studies revealed the cooperative nature of IHF binding at operator sites, whereas the gene expression studies addressed its role in regulating the bacterial motility, stress-related, and glyoxylate shunt genes (22, 25–27). However, much less is known regarding the regulatory roles of IHF in anaerobic fermentative conditions. Apart from the huge number of genes directly or indirectly governed by these regulators, the complex molecular mechanisms underlying the coordination of genes within proteome sectors that facilitate system-wide cellular responses remain uncertain. Besides, the molecular basis of modulation of glucose uptake rate *per se*, as a result of changes in proteome partitions in the event of these global regulator deletions, remains unexplored. To disentangle these regulatory effects on cellular physiology, it would be imperative to reexamine them using proteome allocation and metabolomics lenses.

Here, we sought to interrogate a fundamental question as to how these transcriptional regulators orchestrate the efficient allocation of the proteome share toward metabolic processes and ribosomes that collectively accounts for optimal biomass synthesis under an anaerobic fermentative condition. Therefore, employing a systems biology approach, we investigated the molecular bases underlying the phenotypic shifts of the organism as a result of the deletion of global transcriptional regulators that will provide insights into their significance in coordinating the metabolite pools to foster exponential growth. Furthermore, emphasis on changes in gene expression or activity profiles on their coregulators or local regulators modulating specific pathways highlights their widespread interactions within the transcriptional regulatory network.

In this work, we characterized transcriptomic responses of regulator deletion strains that elucidate direct or indirect control over a nitrogen sensing mechanism, repress unnecessary genes under anaerobic fermentation, and influence the biosynthesis and bottleneck reactions in the central carbon metabolic pathways. These primarily represent the core metabolic proteome essential for rapid growth, and their impairment influences the interplay with the growth rate-dependent ribosomal proteome that in turn perturbs the global metabolome of the organism. By developing an extension to a three-component coarse-grained model (4, 7, 9, 11, 28), we systematically revealed that these global regulators ensure fine-tuned sector-specific proteome distribution. Direct implications of our findings also reinforce how such modulations in growth restrain the glucose uptake via intracellular metabolites, despite no changes in gene expression related to glucose import. Overall, our bottom-up approach serves to elucidate the underlying importance of global regulators on central carbon metabolism in E. coli.

## RESULTS

### Disparate transcriptome profiles of global regulator mutants converge to a common phenomenon of metabolic impairment.

First, to address the transcriptomic changes caused by the disruption of global regulators, namely, FNR, ArcA, and IHF, in E. coli K-12 MG1655 under anaerobic fermentation in glucose minimal medium, we performed a high-coverage RNA sequencing (RNA-seq) experiment around the mid-exponential phase of growth (see Data Set S1 in the supplemental material, FNR_gene_expression, ArcA_gene_expression, and IHF_gene_expression sheets). Preliminary analysis of RNA-seq data reiterated the role of FNR and ArcA regulators as a transcriptional activator and repressor, respectively (18–20) (see Fig. S1A and B). In contrast, the pattern of differentially expressed genes (DEGs) in the Δ*ihf* mutant did not ascertain whether IHF acts either as a transcriptional activator or a repressor.

10.1128/mSystems.00001-21.1 FIG S1 Transcriptome comparison of the regulator mutants compared to WT. (A) Stacked plots showing the number of DEGs in all the mutant strains compared to expression in the WT. (B) Volcano plots of the DEGs for Δ*ihf*, Δ*fnr*, and Δ*arcA* mutants compared to expression in the WT, depicted as adjusted *P* (adj-*P*) values (log10 scale) versus fold change (log2 scale). The downregulated genes are shown in brown, and the upregulated genes are shown in cyan. (C) Enrichment of DEGs under regulation of sigma factors in the Δ*ihf*, Δ*fnr*, and Δ*arcA* mutants compared to WT. The brown bars and the cyan bars indicate the fraction of downregulated and upregulated genes, respectively. Significant increase or decrease is denoted by asterisks: *, *P* < 0.01; **, *P* < 10^−4^. Download FIG S1, TIF file, 2.8 MB. Copyright © 2021 Iyer et al. 2021 Iyer et al. https://creativecommons.org/licenses/by/4.0/ This content is distributed under the terms of the Creative Commons Attribution 4.0 International license.

10.1128/mSystems.00001-21.10 DATA SET S1 Supplementary data set. Download Data Set S1, XLSX file, 0.3 MB. Copyright © 2021 Iyer et al. 2021 Iyer et al. https://creativecommons.org/licenses/by/4.0/ This content is distributed under the terms of the Creative Commons Attribution 4.0 International license.

We compared our gene expression data with previous studies encompassing gene expression and binding profiles (17–20, 29) to evaluate the direct and indirect effects of these transcriptional regulators. We observed good agreement of gene expression data with the available expression data sets (Data Set S1, FNR comparison gene expression, ArcA comparison gene expression, and IHF comparison gene expression sheets). Our data showed a significant yet lower percentage of DEGs directly regulated by FNR (upregulated [up], ∼11%, *P* > 0.05; downregulated [down], ∼14%, *P* < 10^−3^), ArcA (up, ∼30%, *P* < 10^−4^; down, ∼29%, *P* < 10^−4^), and IHF (up, ∼10%, *P* < 10^−4^; down, ∼17%, *P* < 10^−5^). Moreover, DEGs showed enrichment for RNA polymerase with either stress-related sigma factor sigma 38 or nitrogen-related factor sigma 54, apart from the growth-associated sigma factor 70 that targets promoters essential for exponential growth (Fig. S1C). The partitioning of RNA polymerase according to various sigma factors determines the function of growth rate-dependent global machinery (5, 6). Hence, perturbations in patterns of RNA polymerase and associated sigma factors reflected a suboptimal functionality of growth rate-dependent global machinery as an indirect consequence due to the loss of these regulators.

To understand the role of these regulators on key metabolic pathways, DEGs were enriched using the KEGG pathway hierarchical classification (see Fig. S2 to S4). We observed a common enrichment (Benjamini-Hochberg adjusted *P* value < 0.05) of pathways such as amino acid metabolism and transport across all the mutants. Additionally, we observed enrichment of pathways of the TCA cycle and anaplerosis and alternate carbon metabolism (e.g., lipid and steroid metabolism, glycerol, or secondary carbon degradation) in these mutants. As metabolic genes are known to be coregulated by other global or local transcription factors, we examined whether any of the transcription factors were differentially expressed in each of the mutants (Data Set S1, Regs differentially expressed sheet). Several regulators were differentially expressed in each of the mutants compared to the wild type (WT) (Data Set S1, Regs differentially expressed sheet). Additionally, the activity of coregulators was inferred from the transcription factor-gene interaction data (RegulonDB) as well as effector metabolite concentrations in each of the mutants. Apart from altered activity of other global regulators, our data also showed altered activity of the regulators that are known to be associated with nucleotide metabolism (NrdR), amino acid metabolism (ArgR, MetJ, and TrpR), nitrogen metabolism (GlnG and PuuR), alternate carbon metabolism (MalT and UlaR), and sulfur metabolism (CysB) (Data Set S1, Regs enrichment sheets for *fnr*, *arcA* and *ihf*). Altogether, such enrichments underscore the metabolic impairment prevalent as a result of the loss of these global regulators.

10.1128/mSystems.00001-21.2 FIG S2 Voronoi maps showing the metabolic pathways and the genes within each pathway, enriched by KEGG classification in the Δ*ihf* mutant compared to that in the WT. (A) Downregulated metabolic pathways and the genes within each pathway. None of the pathways were found to be significantly downregulated. (B) Upregulated metabolic pathways and the genes within each pathway. Amino acid metabolism (adj-*P* < 0.05) and transport (adj-*P* < 10^−3^) were found to be significantly upregulated. The size of the hexagon within each category is directly proportional to the absolute fold change observed for the genes. The color of the hexagon denotes the specific pathways classified by KEGG. Download FIG S2, TIF file, 2.8 MB. Copyright © 2021 Iyer et al. 2021 Iyer et al. https://creativecommons.org/licenses/by/4.0/ This content is distributed under the terms of the Creative Commons Attribution 4.0 International license.

### Perturbation in nitrogen homeostasis.

**FIG 1:**
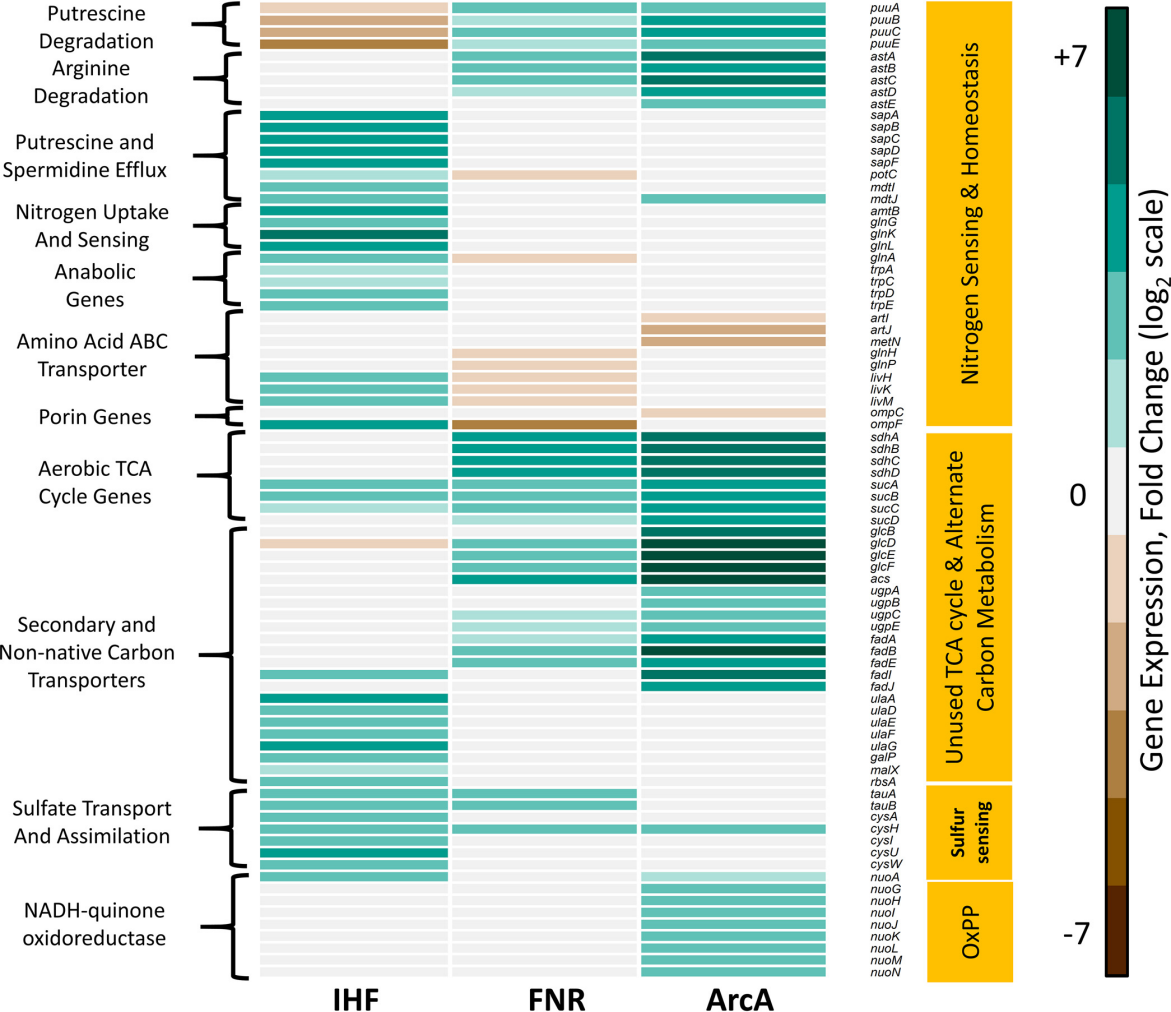
Heat map of representative DEGs in Δ*ihf*, Δ*fnr*, and Δ*arcA* strains, each compared to WT. The figure shows specific genes enriched by KEGG pathway classification (shown in Fig. S2 to S4 in the supplemental material) that were functionally recategorized using evidence from the EcoCyc database. Gene expression values are obtained from the average from two biological replicates (*n* = 2) expressed as log_2_ fold change. The downregulated genes are shown in brown, and the upregulated genes are shown in cyan. Broadly, loss of these global transcriptional regulators was found to commonly affect genes involved in nitrogen sensing and homeostasis, unused TCA cycle and alternate carbon metabolism, sulfur sensing, and aerobic respiration-dependent oxidative phosphorylation (OxPP). We observed changes in outer membrane porin genes, such as downregulation of *ompF* in the Δ*fnr* mutant and *ompC* in the Δ*arcA* mutant and upregulation of *ompF* in the Δ*ihf* mutant, perceived as hyperosmotic or hypo-osmotic conditions, respectively. Alteration of these porin genes reflected the osmotic imbalance faced by the cell, which in turn has strong associations with nitrogen assimilation (43, 81).

We investigated the pivotal gene-level changes associated with the significant pathways commonly enriched in all the mutants (Fig. 1 and Fig. S2 to S4). Specifically, we observed the upregulation of putrescine (*puuABCE*) and arginine degradation (*astABCDE*) genes in Δ*fnr* and Δ*arcA* mutants. These genes belong to pathways that yield glutamate and ammonia as end products that can independently satisfy E. coli’s nitrogen requirement (30–32). Perhaps, upregulation of these genes indicated a scavenging mechanism to restore possible disruption of the nitrogen balance in the cell. The putrescine degradation genes are directly repressed by PuuR, whereas the arginine degradation genes are directly activated by GlnG and ArgR transcription factors, respectively. We observed an increase in PuuR transcript levels but reduced activity as inferred from upregulation of its target genes in the case of Δ*fnr* and Δ*arcA* mutants. On the other hand, we observed an increase in the activity of GlnG and ArgR in both mutants (Data Set S1, Regs enrichment sheets for *fnr* and *arcA*).

A similar scenario of nitrogen limitation was prevalent in the Δ*ihf* mutant (Fig. 1), wherein we observed increased gene expression of ATP-dependent nitrogen uptake (*amtB*) and two-component nitrogen sensing systems (*glnKLG*), which are known to be expressed under nitrogen-replete conditions (30, 33). Besides, we observed the upregulation of amino acid biosynthesis genes such as glutamine (*glnA*) and tryptophan (*trpACDE*) in the Δ*ihf* mutant, in agreement with increased expression of anabolic genes in response to nitrogen limitation (11). Overall, such characteristic expression profiles elucidated an important role of these global regulators in regulating the nitrogen homeostasis and sensing mechanism in E. coli.

### Derepression of genes unnecessary in anaerobic fermentation.

Our analysis indicated an upregulation of the TCA cycle and anaplerosis pathway in Δ*fnr* and Δ*arcA* mutants (Fig. 1 and Fig. S3 and S4) that involved genes coding for the aerobic respiratory TCA cycle, in agreement with previous data sets (18–20, 29). Apart from being unnecessary under the anaerobic fermentative conditions, these genes also incur a substantial protein synthesis cost (34). In addition, we observed a significant upregulation of alternate carbon metabolism genes such as glycolate degradation (*glcBDEF*), *sn*-glycerol-3-phosphate transport (*ugpABCE*), fatty acid degradation (*fadABEIJ*), and acetate uptake (*acs*) in Δ*fnr* and Δ*arcA* mutants. Furthermore, we observed the upregulation of known aerobic oxidative phosphorylation (*nuoAGHIJKLMN*) genes and maltose ABC transporters (*malEFGPKB*) in the Δ*arcA* mutant. Maltose transporters are directly activated by MalT, which was significantly upregulated in the Δ*arcA* mutant. Collectively, this represents a hedging mechanism, wherein a bacterial cell when challenged with fluctuating or unfavorable environmental conditions modulates its gene expression to prepare itself to be conducive to alternate metabolic phenotypes (35–37).

10.1128/mSystems.00001-21.3 FIG S3 Voronoi maps showing the metabolic pathways and the genes within each pathway, enriched by KEGG classification in the Δ*fnr* mutant compared to that in the WT. (A) Downregulated metabolic pathways and the genes within each pathway. Transport (adj-*P* < 0.002) was found to be significantly downregulated. (B) Upregulated metabolic pathways and the genes within each pathway. Amino acid metabolism (adj-*P* < 10^−3^) and TCA cycle (adj-*P* < 10^−8^) were found to be significantly upregulated. The size of the hexagon within each category is directly proportional to the absolute fold change observed for the genes. The color of the hexagon denotes the specific pathways classified by KEGG. Download FIG S3, TIF file, 2.3 MB. Copyright © 2021 Iyer et al. 2021 Iyer et al. https://creativecommons.org/licenses/by/4.0/ This content is distributed under the terms of the Creative Commons Attribution 4.0 International license.

10.1128/mSystems.00001-21.4 FIG S4 Voronoi maps showing the metabolic pathways and the genes within each pathway, enriched by KEGG classification in the Δ*arcA* mutant compared to that in the WT. (A) Downregulated metabolic pathways and the genes within each pathway. Amino acid metabolism (adj-*P* < 10^−3^) and transport (adj-*P* < 10^−3^) were found to be significantly downregulated. (B) Upregulated metabolic pathways and the genes within each pathway. Amino acid metabolism (adj-*P* < 0.0152), TCA cycle (adj-*P* < 10^−10^), transport (adj-*P* < 0.012), oxidative phosphorylation (adj-*P* < 10^−5^), and lipid metabolism (adj-*P* < 10^−3^) were found to be significantly upregulated. The size of the hexagon within each category is directly proportional to the absolute fold change observed for the genes. The color of the hexagon denotes the specific pathways classified by KEGG. Download FIG S4, TIF file, 2.4 MB. Copyright © 2021 Iyer et al. 2021 Iyer et al. https://creativecommons.org/licenses/by/4.0/ This content is distributed under the terms of the Creative Commons Attribution 4.0 International license.

Similarly, the Δ*ihf* mutant showed significant upregulation of alternate carbon metabolism genes responsible for ascorbate (*ulaADEFG*), galactose/maltose (*galP*, *malX*), and ribose (*rbsA*) catabolism (Fig. 1). We observed lowered activity of UlaR, which is responsible for complete repression of ascorbate degradation genes in conjunction with IHF (38). We further observed the upregulation of genes coding for transporters of sulfur sources (*tauAB* and *cysAUW*) as well as sulfate assimilation (*cysHI*) in conjunction with increased activity of CysB regulator in the Δ*ihf* mutant. Overall, the upregulation of genes of unused or hedging proteins under conditions of anaerobic fermentation can impose a burden on the growth of the organism.

### Regulatory control of amino acid biosynthesis and bottleneck steps in the central carbon metabolic pathway.

To effectively examine the control of amino acid biosynthesis and specific bottleneck reactions, we monitored the absolute intracellular concentrations of 40 metabolites of central carbon metabolism in the mid-exponential phase using ^13^C-based metabolomics (Data Set S1, Abs_conc of metabolites sheet) coupled to the corresponding gene expression changes. We examined the changes in absolute metabolite concentrations in each mutant compared to the WT, and only those metabolites which were found to be significantly altered (false-discovery rate [FDR] < 0.05) were further analyzed.

**FIG 2:**
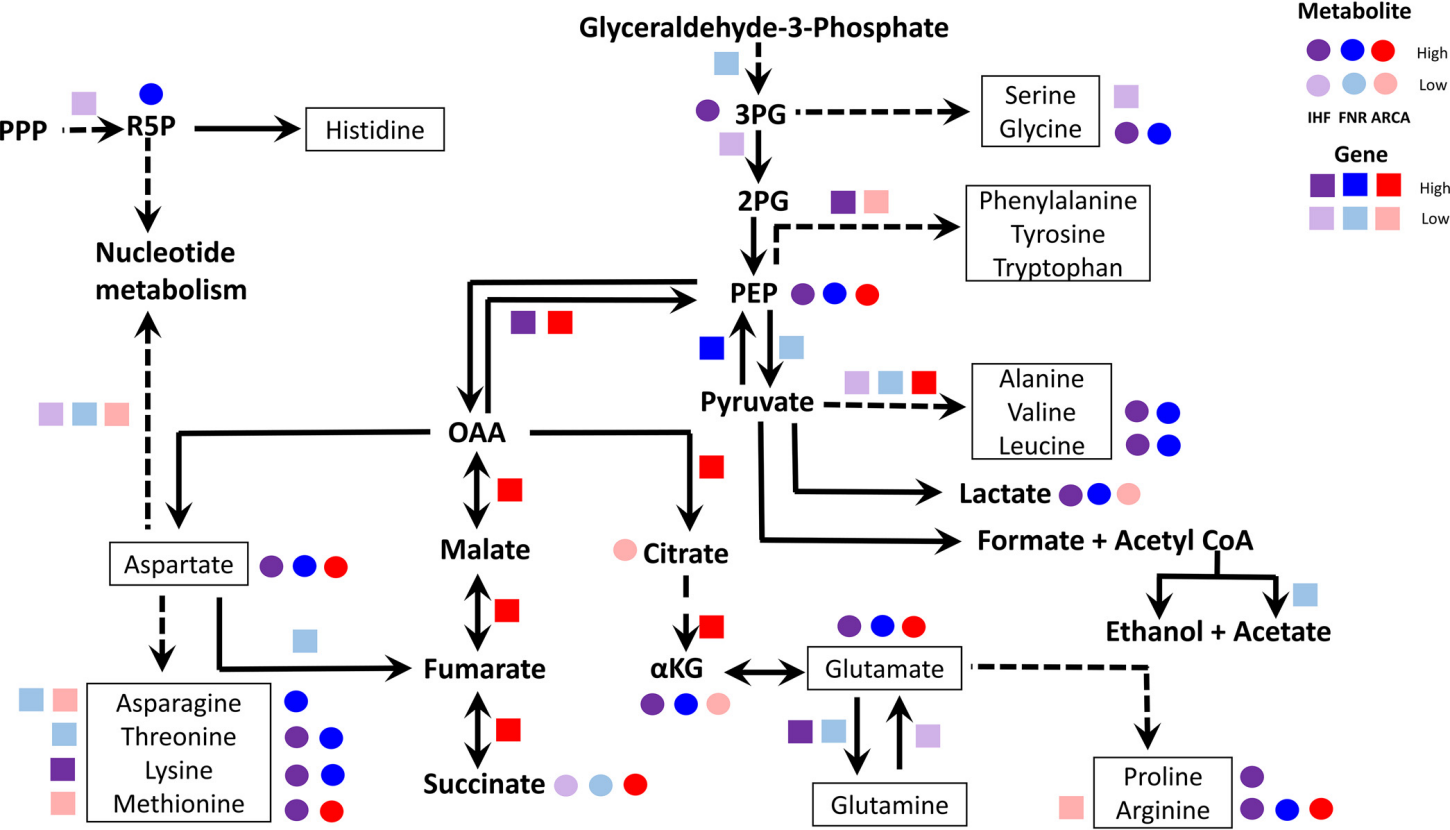
Schematic representation of the key steps in central carbon metabolic pathways affected by the deletion of either of the global regulators IHF, FNR, and ArcA. The figure shows the unique pattern of regulatory control exerted on gene expression as well as metabolite levels of amino acid biosynthesis and rate-limiting steps in glycolytic reactions. Apart from control of the oxidative and reductive arm of the TCA cycle, our data indicate the downregulation of nucleotide biosynthesis genes in all the mutants, which succinctly explains the regulatory control of purine and pyrimidine metabolism. The genes are represented as colored squares, and metabolites are represented as colored circles. The colors purple, blue, and red represent changes in gene expression (squares) or metabolite levels (circles) in Δ*ihf*, Δ*fnr*, and Δ*arcA* mutants compared to WT, respectively. The dashed lines indicate two or more reactions connecting the metabolites. Darker colors represent upregulation, whereas light colors represent downregulation. Note that succinate and lactate represent exometabolite yields and are not measured intracellularly. Only statistically significant gene expression and metabolite levels are depicted. Abbreviations: 3PG, glycerate-3-phosphate; 2PG, glycerate-2-phosphate; PEP, phosphoenolpyruvate; OAA, oxaloacetate; αKG, α-ketoglutarate; PPP, pentose phosphate pathway; R5P, ribose-5-phosphate.

**FIG 3:**
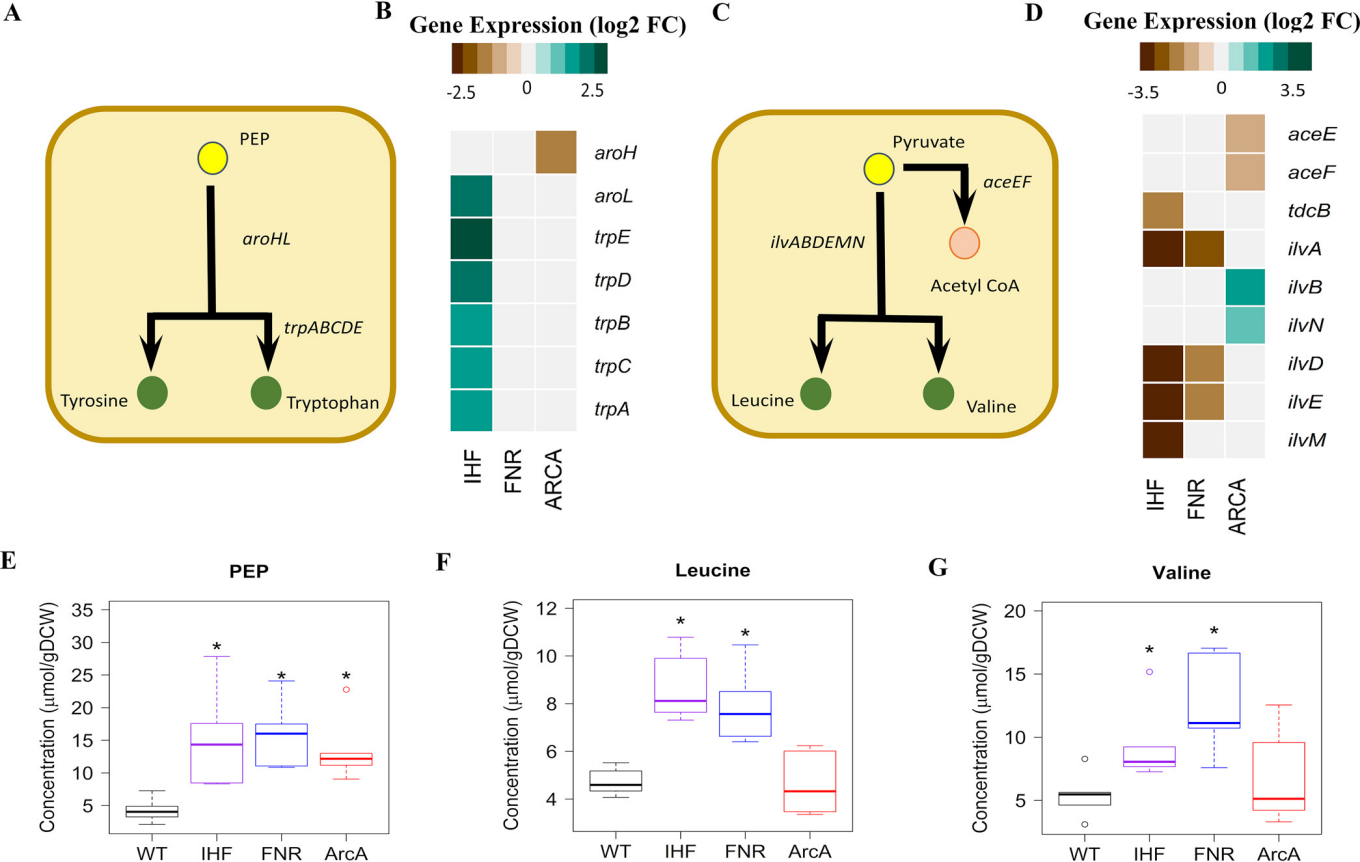
Integrated transcriptomics and metabolomics analysis at PEP and pyruvate node. (A and C) Ball and stick representations of pathways annotated with genes were obtained from EcoCyc, wherein yellow color represents the precursors PEP and pyruvate, and green color represents amino acids. (B and D) Gene expression profiles of DEGs altered in the pathways depicted as heat map were obtained by comparing each of the regulator mutants (Δ*ihf*, Δ*fnr*, and Δ*arcA*) with WT. Expression values were obtained from the average from two biological replicates (*n* = 2) expressed as log_2_ fold change. (E to G) Metabolite concentrations were obtained from an average of three biological and two technical replicates (*n* = 6) expressed as micromoles per gram (dry cell weight) (μmol/gDCW). Only those metabolites which are significant at least in one mutant compared to WT are projected. *, statistically significant metabolite in each mutant compared to WT. Open colored circles represent outliers for each of the mutants. The list of the measured metabolites and their absolute concentrations are given in Data Set S1 (List of metabolites and Abs_conc of metabolites sheets).

Among the precursor metabolites of the glycolytic pathway, a pronounced increase in phosphoenolpyruvate (PEP) accumulation was seen in all the mutants (Fig. 2 and 3E). Presumably, this observation can be attributed to the upregulation of *pck* in Δ*ihf* and Δ*arcA* mutants and upregulation of *ppsA* and downregulation of *pykA* genes in the Δ*fnr* mutant (see Fig. S5C). The gluconeogenic *pck* and *ppsA* genes have been reported to contribute to PEP synthesis during glycolysis from oxaloacetate (OAA) and pyruvate, respectively (39). On the other hand, the *pykA* gene, involved in the conversion of PEP to pyruvate with the generation of ATP by substrate-level phosphorylation, represents an important rate-limiting enzyme under anaerobic fermentative conditions (40). Despite an increase in PEP levels and changes in gene expression of biosynthesis of aromatic amino acids (tyrosine, tryptophan, and phenylalanine), we did not observe a concomitant increase in their levels in any of the mutants.

10.1128/mSystems.00001-21.5 FIG S5 Integrated transcriptomics and metabolomics analysis at 3PG and R5P node. (A) Ball and stick representation of the pathway annotated with genes is obtained from EcoCyc, wherein yellow color represents the precursor 3PG and R5P and green color represents amino acids. (B) Ball and stick representation of the transhydrogenase reaction annotated with genes is obtained from EcoCyc. (C) Expression profile of DEGs altered in the pathways depicted as a heat map was obtained by comparing each of the regulator mutants (Δ*ihf*, Δ*fnr*, and Δ*arcA*) with WT. Gene expression values were obtained from the average from two biological replicates (*n* = 2) expressed as log2 fold change. (D to G) Metabolite concentrations were obtained from the average from three biological and two technical replicates (*n* = 6) expressed as micromoles per gram (dry cell weight) (μmol/gDCW). Only those metabolites which are significant at least in one mutant compared to WT are projected. *, statistically significant metabolite in each mutant compared to WT. The list of the measured metabolites and their absolute concentrations are given in Data Set S1 (List of metabolites and Abs_conc of metabolites sheets). Download FIG S5, TIF file, 2.5 MB. Copyright © 2021 Iyer et al. 2021 Iyer et al. https://creativecommons.org/licenses/by/4.0/ This content is distributed under the terms of the Creative Commons Attribution 4.0 International license.

Besides, we observed the accumulation of branched-chain amino acids derived from pyruvate, namely, leucine and valine, in Δ*ihf* and Δ*fnr* mutants (Fig. 3F and G). This aligned with lowered gene expression of *ilvADEM* observed for both the mutants (Fig. 3D), thereby indicating a feedback regulation of these amino acids on their biosynthesis. On the contrary, we observed increased gene expression of *ilvB* and *ilvN* genes which catalyze the first step in branched-chain amino acid synthesis, without changes in leucine and valine levels in the case of the Δ*arcA* mutant. Additionally, in the Δ*ihf* mutant, we observed an increase in levels of glycerate-3-phosphate (3PG), a precursor for the amino acids glycine and serine, in concert with downregulation of *gpmA* gene expression (Fig. 2 and Fig. S5E). However, only glycine levels were found to be higher in both Δ*ihf* and Δ*fnr* mutants (Fig. S5D). Such PEP and 3PG accumulations are known to signal carbon limitation (41), and their downstream amino acids with dysregulated patterns are known to have occurred in other stresses as well (42).

**FIG 4:**
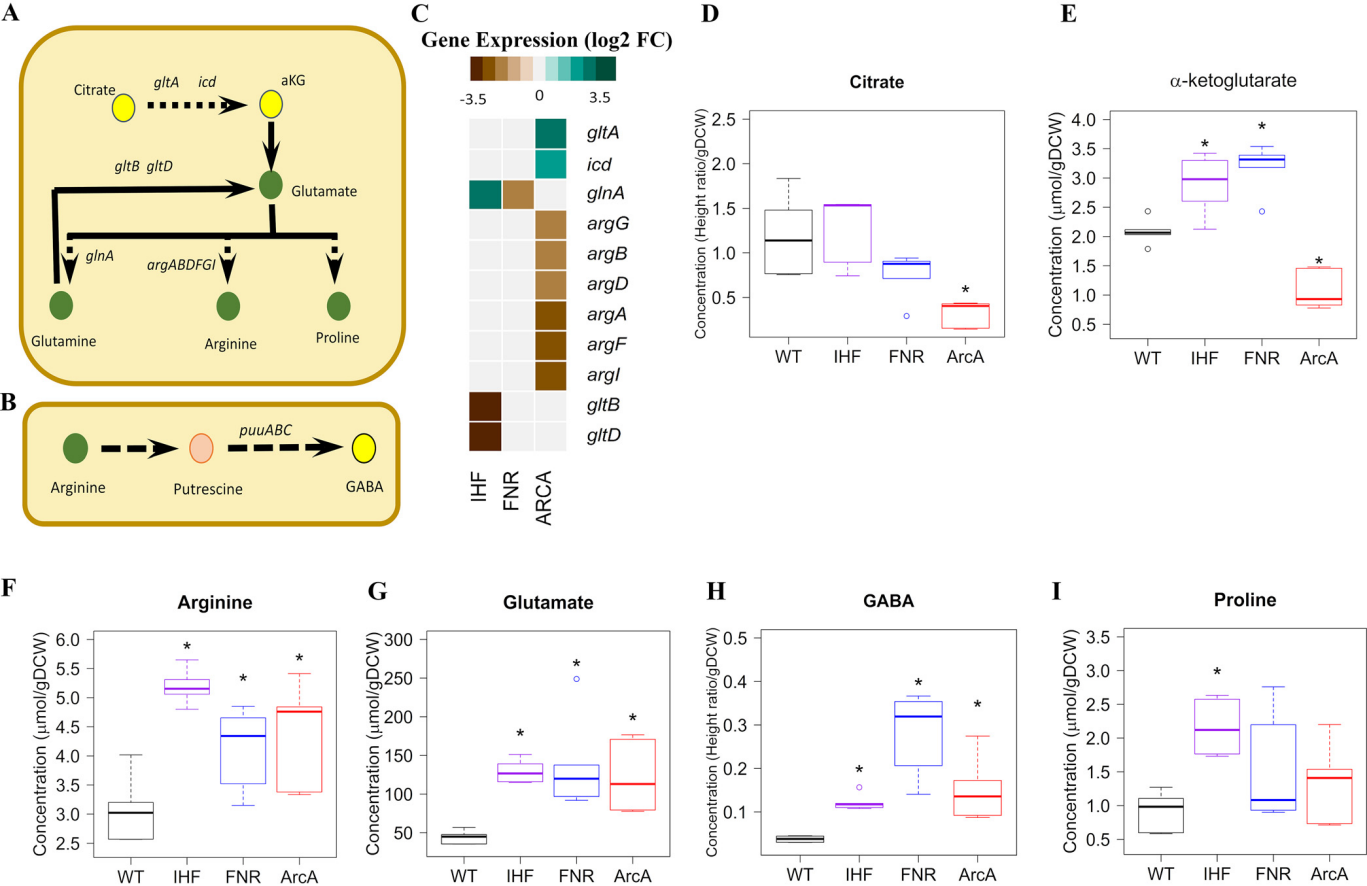
Integrated transcriptomics and metabolomics analysis at citrate and αKG node. (A) Ball and stick representation of the pathway annotated with genes was obtained from EcoCyc, wherein yellow color represents the precursors citrate and αKG and green color represents amino acids. (B) Ball and stick representation of the arginine and putrescine degradation pathway annotated with genes was obtained from EcoCyc, wherein yellow color represents GABA, and green color represents the amino acid arginine. (C) Expression profiles of DEGs altered in the pathways depicted as a heat map obtained by comparing each of the regulator mutants (Δ*ihf*, Δ*fnr*, and Δ*arcA*) with WT. Gene expression values were obtained from an average from two biological replicates (*n* = 2) expressed as log_2_ fold change. (D to I) Metabolite concentrations were obtained from an average from three biological and two technical replicates (*n* = 6) expressed as micromoles per gram (dry cell weight) (μmol/gDCW). Only those metabolites which are significant at least in one mutant compared to WT are projected. *, statistically significant metabolite in each mutant compared to WT. Open colored circles represent outliers for each of the mutants. The list of the measured metabolites and their absolute concentrations are given in Data Set S1 (List of metabolites and Abs_conc of metabolites sheets).

Next, we examined the TCA cycle intermediate α-ketoglutarate (αKG), which coordinates carbon and nitrogen balance by modulating glycolytic flux and accumulates during nitrogen limitation (43, 44). Indeed, both Δ*ihf* and Δ*fnr* mutants showed an accumulation of αKG in contrast to the Δ*arcA* mutant, which showed a reduction (Fig. 2 and 4E). It is quite surprising that citrate (Fig. 4D) and αKG levels were lower in the Δ*arcA* mutants despite the upregulation of *gltA* and *icd* genes, which might indicate reduced TCA cycle activity (45, 46).

**FIG 5:**
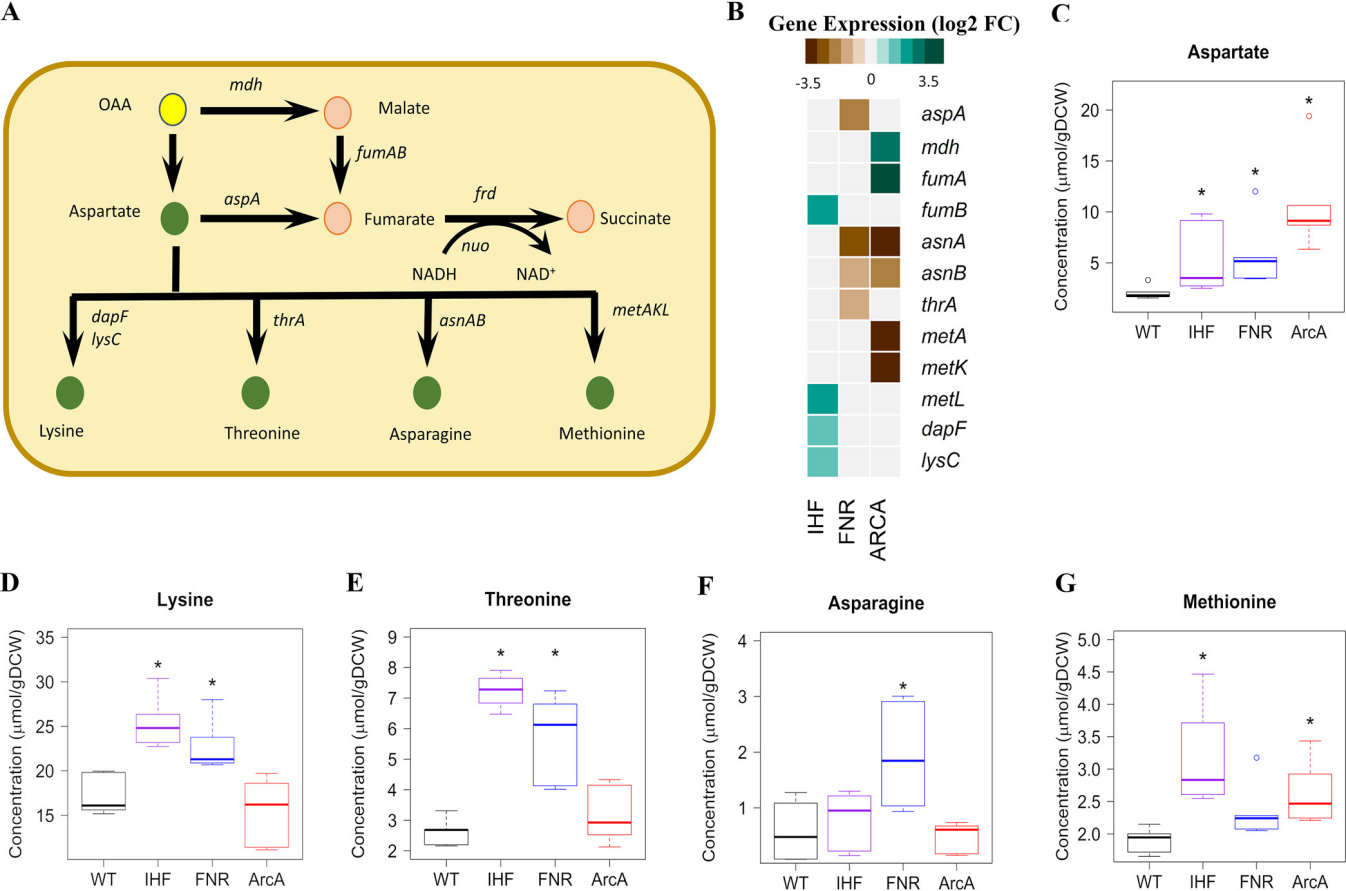
Integrated transcriptomics and metabolomics analysis at OAA node. (A) Ball and stick representation of the pathway annotated with genes is obtained from EcoCyc, wherein yellow color represents the precursor OAA and green color represents amino acids. (B) Gene expression profile of DEGs altered in the pathways depicted as a heat map was obtained by comparing each of the regulator mutants (Δ*ihf*, Δ*fnr*, and Δ*arcA*) with WT. Expression values were obtained from an average from two biological replicates (*n* = 2) expressed as log_2_ fold change. (C to G) Metabolite concentrations were obtained from an average from three biological and two technical replicates (*n* = 6) expressed as micromoles per gram (dry cell weight) (μmol/gDCW). Only those metabolites which are significant at least in one mutant compared to WT are projected. *, statistically significant metabolite in each mutant compared to WT. Open colored circles represent outliers for each of the mutants. The list of the measured metabolites and their absolute concentrations are given in Data Set S1 (List of metabolites and Abs_conc of metabolites sheets).

The amino acids derived from αKG, namely, glutamate, proline, and arginine, as well as the amino acids derived from OAA, namely, aspartate, lysine, methionine, and threonine, were found to be significantly higher in Δ*ihf* and Δ*fnr* mutants (Fig. 2, 4F to I, and 5C to G). Arginine is known as a positive coeffector of ArgR activity. Hence, the increased ArgR activity based on the increased expression of arginine degradation genes (Fig. 1) could be attributed to high internal arginine levels despite the *argR* gene itself being downregulated in the Δ*fnr* mutant. Similarly, the reduced expression of glutamate degradation genes (*gltB* and *gltD*) (Fig. 4C) could be attributed to increased ArgR activity despite the *argR* gene itself being downregulated in the Δ*ihf* mutant (Data Set S1, Regs enrichment sheets for *ihf*). In the Δ*arcA* mutant, concomitant with an increase in aspartate and glutamate levels, we observed increased accumulation of methionine and arginine, respectively. The changes in their levels were in line with the downregulation of methionine (Fig. 5B) and arginine biosynthesis genes (Fig. 4C), suggesting feedback repression mediated through MetJ and ArgR, respectively. Since arginine degradation and methionine or lysine biosynthesis results in the synthesis of intracellular ammonia and succinate (31, 47), higher pools of these amino acids possibly indicate a strategy to circumvent nitrogen limitation. Additionally, the increased putrescine accumulation in response to nitrogen limitation can be inferred from the increased levels of arginine and gamma-aminobutyric acid (GABA) (Fig. 4H) observed in all the mutants. Hence, the increased expression of putrescine degradation genes (*puuABCE*) (Fig. 1), despite an increase in the expression of *puuR* itself observed in the Δ*fnr* and Δ*arcA* mutants, could be explained by the inhibitory effect of putrescine on PuuR activity. However, we observed lowered expression of the putrescine degradation genes (Fig. 1) concomitant with increased PuuR activity in the case of the Δ*ihf* mutant, despite higher putrescine levels.

Our data also indicate the downregulation of nucleotide biosynthesis genes in all the mutants (Fig. S5C), which explains their regulatory control on purine and pyrimidine metabolism. This could be partly explained by the increased activity of NrdR. As nucleotide biosynthesis, akin to that of amino acids, involves feedback repression, together with an observed increase in precursor aspartate levels (Fig. 2), we speculate that higher levels of deoxyribonucleotide triphosphates might have increased the activity of NrdR (48) in each of the mutants.

Overall, we observed that increased PEP levels were mirrored as increased OAA (inferred from aspartate pools) and pyruvate levels (inferred from leucine and valine pools) in the cases of Δ*ihf* and Δ*fnr* mutants and solely as increased OAA levels in the case of the Δ*arcA* mutant, without any concomitant increase in the costly aromatic amino acid levels in any of the mutants. Second, changes in amino acid pools as a result of increased degradation or biosynthesis of amino acids derived from aspartate or glutamate represent resources for the generation of intracellular ammonia and succinate. Nevertheless, such amino acid accumulations in all the mutants could be, in part, due to the inability of the organism to efficiently utilize them toward protein biomass essential for optimal growth. To summarize, we observed direct and indirect effects on the metabolome due to the deletion of global regulators, which corroborated the upset in gene expressions in amino acid biosynthesis and other bottleneck reactions.

### Physiological characterization highlights the growth suboptimality.

Severe perturbation in metabolic processes due to the deletion of regulators motivated us to examine their physiological effects on the organism. Our data showed a profound effect on growth physiology, glucose, and nitrogen import, thereby elaborating on the system-wide effect of the deletion of global regulators under anaerobic fermentation of glucose. Characterization of the phenotype of the mutants revealed a reduction of ∼16% to 25% (*P* < 0.05, Student’s *t* test) in the growth rate as well as glucose uptake rate compared to those of the WT (see Table S1). The decrease in growth rate was found to be positively correlated with a decrease in glucose uptake rate (Pearson correlation coefficient [PCC], ∼0.98, *P* < 10^−3^) (see Fig. S6A to C). As PEP is known to donate a phosphate group to the EI (enzyme I) protein of the phosphotransferase system, thereby facilitating the uptake of glucose (49), the decrease in glucose uptake was in agreement with the accumulation of PEP observed across the mutants (PCC, ∼−0.9, *P* < 0.05) (Fig. S6A to C).

10.1128/mSystems.00001-21.6 FIG S6 Pairwise Pearson correlation plots of log2-normalized metabolite concentrations as well as glucose uptake rate (Glc_up) and growth rate (GR) for Δ*ihf* mutant with WT (A), Δ*fnr* mutant with WT (B), and Δ*arcA* mutant with WT (C). Only the significantly altered metabolites in each mutant compared to WT were considered for the analysis. Succinate and lactate represent log2-normalized exometabolite yields. In the figure, αKG is denoted as oxoglutarate and ribose-5-phosphate is denoted as R5P. Download FIG S6, TIF file, 2.8 MB. Copyright © 2021 Iyer et al. 2021 Iyer et al. https://creativecommons.org/licenses/by/4.0/ This content is distributed under the terms of the Creative Commons Attribution 4.0 International license.

10.1128/mSystems.00001-21.8 TABLE S1 Physiological characterization of the strains in anaerobic fermentation of glucose. The measurements of growth rate, glucose and ammonia uptake rate, and yields of mixed-acid fermentation products were obtained from three biological replicates (*n* = 3). Yields were calculated by normalizing secretion rates with its glucose uptake rate. The errors indicate standard deviations within the replicates. Download Table S1, DOCX file, 0.01 MB. Copyright © 2021 Iyer et al. 2021 Iyer et al. https://creativecommons.org/licenses/by/4.0/ This content is distributed under the terms of the Creative Commons Attribution 4.0 International license.

Furthermore, we measured fermentation products or exometabolites, namely, formate, acetate, ethanol, and lactate, arising from pyruvate and succinate arising from PEP (Table S1). Our findings of increased succinate yield in the Δ*arcA* mutant and reduced succinate yield in the Δ*fnr* mutant were consistent with previous studies (20, 29), without any distinct association with the trends of growth rates or glucose uptake rates. Additionally, we obtained higher lactate yield in the Δ*fnr* mutant, which had strong correlations (PCC, ∼0.95, *P* < 0.005) with branched-chain amino acids, namely, leucine and valine, both being derivatives of pyruvate (Fig. S6A to C). The branched-chain amino acids leucine and valine showed a significant increase in the case of the Δ*ihf* mutant as well. Together with a higher yet insignificant lactate yield observed in the case of the Δ*ihf* mutant, we anticipated slightly higher intracellular pyruvate levels in the mutant similar to that in the Δ*fnr* mutant. However, lower but significant lactate yields accompanied with insignificant changes in leucine and valine in the case of the Δ*arcA* mutant reveals an antagonistic control of ArcA on the pyruvate node compared to that of FNR and IHF.

Next, we measured the ammonia uptake rate as an indicator of the nitrogen status of the organism. All the mutants showed a reduced ammonia uptake rate (*P* < 0.05, Student’s *t* test) compared to that of the WT (Table S1), reflecting the nitrogen limitation faced by the organism, congruent with our gene expression and metabolite data (αKG, arginine, and glutamate).

### Global transcriptional regulators control the translational and metabolic efficiency of the organism.

By exploiting the gene expression and intracellular and exometabolite profiles, we sought to quantitatively examine these changes at the proteome level that correspond to impaired biomass synthesis or growth rate and subsequent glucose uptake. Consequently, we recalled a proteome allocation model proposed by the Hwa group (4, 7, 9, 11, 28) that quantitatively relates the cellular ribosome and metabolic proteome contents to the growth of E. coli. We substantiate our key findings by defining a four-partition model that projects the major subsystems, namely, R (ribosome), M (metabolic), and U (unnecessary) sectors proportional to our experimental measurements with a fixed core Q sector.

Proteome sectors (∅) comprise the following: an R sector, defining the growth rate-dependent ribosomal sector; M sector, defining the growth rate-dependent metabolic protein sector; and Q sector, defining the growth rate-independent core proteome sector (9). Q sector represents the proteome belonging to replication and cell membrane biogenesis, etc., which are maintained at constant proportions by autonegative feedback regulation as they are essential for the growth of the organism (7, 9). We defined an additional neutral proteome sector “U” for the unused or unnecessary metabolic proteins that help the organism hedge against unfavorable environmental conditions but presumably pose a substantial proteomic burden under anaerobic fermentative conditions. These U sector proteins defined here do not account for exogenously expressed proteins but rather endogenous proteins that can switch to being necessary when subjected to other nutritional or respiratory conditions. However, these U sector proteins reduce the proteome share available for R and M sectors, effectively reducing the growth rate in analogy to the induced expression of exogenous proteins, devoid of any phenomenological parameters (9). Based on the proteome conservation law, all the aforementioned sectors add up to unity:

(1) ∅R + ∅M + ∅U + ∅Q=1

wherein the growth-related total proteome fraction ($\emptyset_{\max}$) comprises the sum of R, M, and U sectors other than ∅Q,

(2) $$\;\emptyset_{\max}=\emptyset \text{R }+\;\emptyset \text{M }+\;\emptyset \text{U. }$$

Therefore,

$$\emptyset_{\max}=1\;-\;\emptyset \text{Q.}$$

**FIG 6:**
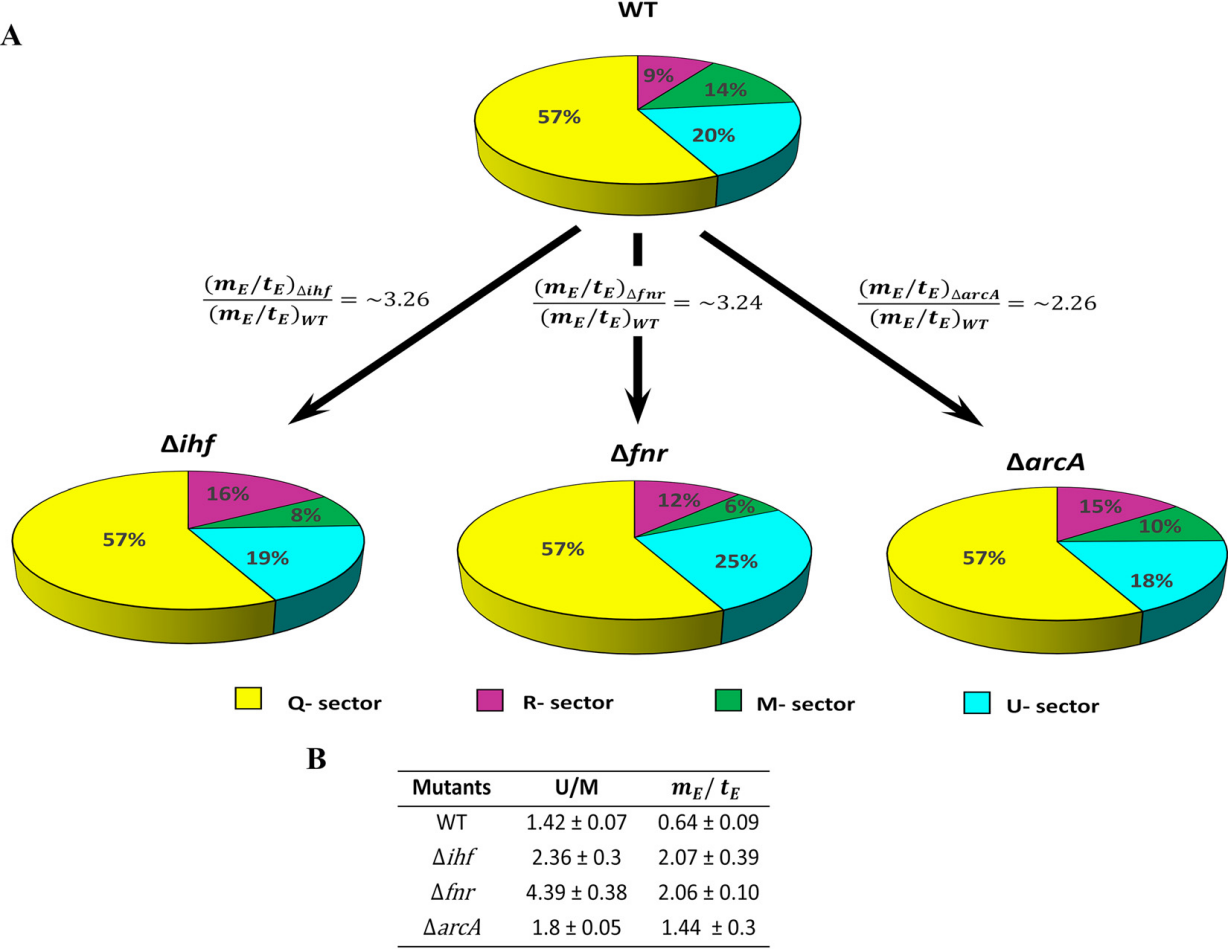
Proteome allocation in each of the strains. (A) Pie chart depicting the quantitative distribution of the proteome sector fractions, R sector (∅R), M sector (∅M), Q sector (∅Q), and U sector (∅U), as percentages for the WT and the mutants. The figure indicates a high $\frac{m_{E}}{t_{E}}$ ratio represented as fold changes in each mutant compared to that for the WT. (B) The U/M protein-coding transcriptome fraction ratio and $\frac{m_{E}}{t_{E}}$ ratio for each of the strains with standard deviations of biological duplicates.

The R sector was derived from the product of experimentally measured total RNA/total protein (R/P) ratio and empirically derived conversion factor ρ = 0.76 (∅R = R/P × ρ), obtained from reference 9, in each of the strains (Fig. 6A). This traditional method of relative ribosome measurement has been reported to be in quantitative agreement with mass spectrometry or β-galactosidase assay data (28). All the mutants in our study showed a higher R/P value than the WT (see Fig. S7), with the Δ*ihf* mutant showing the highest R/P. Notably, there exists a strong correlation between high ribosome content and lowered ppGpp levels (7). Interestingly, our analysis supports this notion in the case of the Δ*ihf* mutant, wherein DEGs that were reported to be positively regulated by ppGpp were downregulated (*P* < 10^−4^, Fisher’s exact test), possibly suggesting a lowered level of ppGpp in this mutant. This observation also reiterated the previously deduced overlap in ppGpp and IHF targets (24). However, such enrichments were found to be insignificant in the cases of Δ*fnr* and Δ*arcA* mutants. Studies have shown that despite any obvious enrichments observed for ppGpp, the R sector could be modulated by amino acid pools directly or by adjusting the expression of rRNA genes by their constitutive promoters in exponentially growing cells (3, 4, 10, 12). The increase in R sector reflects a compensatory response to ameliorate the synthesis of metabolic proteins to maximize biomass synthesis rate. However, R sector synthesis *per se* incurs a huge proteomic cost that reduces the proteome resources available to the M sector. Thus, assuming WT has an optimal R sector given the maximum growth rate, we consider that any excess above the optimal R sector accounts for unused or unnecessary ribosomes.

10.1128/mSystems.00001-21.7 FIG S7 Total RNA/total protein ratio (R/P). Bar plot depicting the experimentally derived total RNA/total protein ratios in all the strains (WT and Δ*ihf*, Δ*fnr*, and Δ*arcA* mutants) depicted as microgram per milligram (dry cell weight) total RNA/microgram per milligram (dry cell weight) total protein. Error bars represent standard deviations within biological replicate samples (*n* = 2). Download FIG S7, TIF file, 0.8 MB. Copyright © 2021 Iyer et al. 2021 Iyer et al. https://creativecommons.org/licenses/by/4.0/ This content is distributed under the terms of the Creative Commons Attribution 4.0 International license.

To evaluate the corresponding changes in M sector, we first performed a simulation specifically under glucose fermentative conditions using the genome-scale metabolism and macromolecular expression (ME) model that accounts for 80% of the E. coli proteome (50, 51). From the simulation, we predicted the set of protein-coding genes (Data Set S1, ME_nonME_genes_anaerobic sheet) for glucose fermentative conditions and subsequently utilized them to enrich the combined list of DEGs obtained from all the mutants (Data Set S1, Transcriptome fractions sheet) that collectively accounted for M and U sector genes. These enriched genes were utilized to calculate the unnecessary/metabolic (U/M) ratio using the protein-coding transcriptome fractions computed for each of the strains (Data Set S1, Transcriptome fractions sheet) (Fig. 6B). As expected, we observed a consistent increase in the U/M ratio across the mutants compared to that for the WT, with the highest for the Δ*fnr* mutant followed by Δ*ihf* and Δ*arcA* mutants. We assumed this transcriptome fraction proportional to its proteome fraction as a reasonable approximation at the subsystem (M or U sector) or functional categorical level rather than individual mRNA-to-protein relation for each gene (10, 28, 52). The disruption in M sector and U sector proteomes reflects the impairment in the protein economy in the mutants. Finally, using the $\emptyset_{\max}$ value (∼43%) reported in literature (7, 11), ∅R fraction, and U/M ratio, we determined the ∅M fraction as defined in equation 2.

For steady-state growth, the R sector and M sector proteome fractions have a linear dependency on growth rate (7, 9), described as:

(3) $$\text{λ}=t_{E}\emptyset \text{R,}$$

(4) $$\text{λ}=m_{E}\emptyset \text{M,}$$

where the phenomenological parameter $t_{E}$ refers to translation efficiency (protein synthesis flux by R sector proteome), phenomenological parameter $m_{E}$ refers to metabolic efficiency (metabolic flux attained by M sector proteome) and λ refers to the growth rate (h^−1^) of the organism.

Hence, using experimentally derived growth rates, R sector proteome fraction, and the derived M sector proteome fraction, we computed the translational and metabolic efficiency in the mutants to quantify the extent of the regulatory defect. By equating equations 3 and 4, we obtain the ratio of metabolic efficiency to translation efficiency.

(5) $$\frac{m_{E}}{t_{E}}=\frac{\emptyset \text{R}}{\emptyset \text{M}}.$$

Intriguingly, the deletion of global regulators increased $\frac{m_{E}}{t_{E}}$ ratio, indicating a decrease in translation and an increase in metabolic flux (Fig. 6A and B). By normalizing the $\frac{m_{E}}{t_{E}}$ ratio for the Δ*ihf*, Δ*fnr*, and Δ*arcA* mutants with the WT ratio, we observed concordant increases of ∼3.26-, ∼3.24-, and ∼2.26-fold, respectively, that succinctly quantify the significance of these regulators toward biomass synthesis in a WT strain (Fig. 6A). Moreover, the lowered $t_{E}$ and high $m_{E}$ as a result of an imbalance in resource allocation further highlighted the inefficiencies of the growth-related physiological processes (RNA polymerase, ribosome, and metabolites ppGpp or cAMP) that enable internal adjustments to counter the limitation in the absence of these global regulators.

### Physiological consequences of altered *m*_*E*_ and *t*_*E*_ on glucose uptake rates.

Since protein levels are subjected to posttranscriptional and posttranslational regulation (1), we focused on the intracellular metabolite concentration that overall reflects the sum of the activity of metabolic proteins and ribosomes to describe the final physiological state (12, 53). The amino acids and precursor pool sizes were used as a proxy to describe the changes in $t_{E}$ and $m_{E}$ flux of the organism, given their linear negative association (Fig. S6A to C) with growth rate and glucose uptake rate. The accumulation of amino acids glutamate, aspartate, and their derivatives observed consistently in the mutants owing to their inability to be incorporated into protein biomass can be attributed to the reduction in $t_{E}$. Similarly, the increase in $\;m_{E}$, implying an increase in synthesis to meet the reduced translational capacity, was indicated by accumulation of precursors namely, PEP, αKG, OAA (speculated from aspartate levels), and their downstream amino acids in the mutants.

Next, we probed how steady-state metabolite accumulations subjected to changes in growth rate might affect glucose uptake rates in each of the mutants. In Δ*fnr* and Δ*ihf* mutants, the intracellular concentration of αKG, when normalized to cell volume (2.3 μl/mg, obtained from reference 54), matches with its *K_i_* (inhibition constant) value of 1.3 ± 0.1 mM for *ptsI* inhibition. Thus, the higher intracellular concentration of αKG observed in Δ*fnr* and Δ*ihf* mutants can reduce the glucose uptake (PCC, ∼−0.9, *P* < 0.05) by noncompetitive inhibition on the phosphotransferase system (PTS) (44, 55). Similarly, OAA (speculated from aspartate levels), known to modestly inhibit glucose uptake (55), was found to be present at higher concentrations in all three mutants, with the Δ*arcA* mutant having the highest aspartate accumulation (PCC, ∼−0.9, *P* < 0.05). Furthermore, from the accumulation of leucine (PCC, ∼−0.94, *P* < 0.01) and valine (PCC, ∼−0.84, *P* < 0.05), increased lactate yield (PCC, ∼−0.92, *P* < 0.01), and higher $\;m_{E}$ (Fig. 6B), we predict a higher intracellular pyruvate pool in the Δ*fnr* mutant, which is known to adversely impact glucose import (55). Moreover, it is known that the degradation of carbon-rich amino acids such as aspartate and glutamate to their respective α-keto acids can affect the glucose uptake rate (55). Collectively, these observations could, in part, explain why the Δ*arcA* mutant showed slightly better glucose uptake than the Δ*fnr* and Δ*ihf* mutants. Additionally, the extent of reduction in glucose uptake in each of the mutants corroborated the reduction in M sector and increase in U sector (Fig. 6B) as well as the increase in unused R sector, given that glucose import and processing under a minimal medium condition involves significant metabolic proteome resources (56).

## DISCUSSION

Scrupulous proteome allocation in response to perturbations in the internal or external environment defines the growth physiology of E. coli (1–4, 56). Anaerobic fermentation represents an energetically less favorable environmental condition characterized by high carbon uptake and slow growth, wherein an efficient proteome allocation toward energy and biomass becomes imperative (31). In this study, we addressed how global transcriptional regulators FNR, ArcA, and IHF ensure efficient proteome allocation toward biomass synthesis in a WT strain that enables it to attain an economical phenotypic outcome or foster robustness even under such unfavorable environmental conditions.

**FIG 7:**
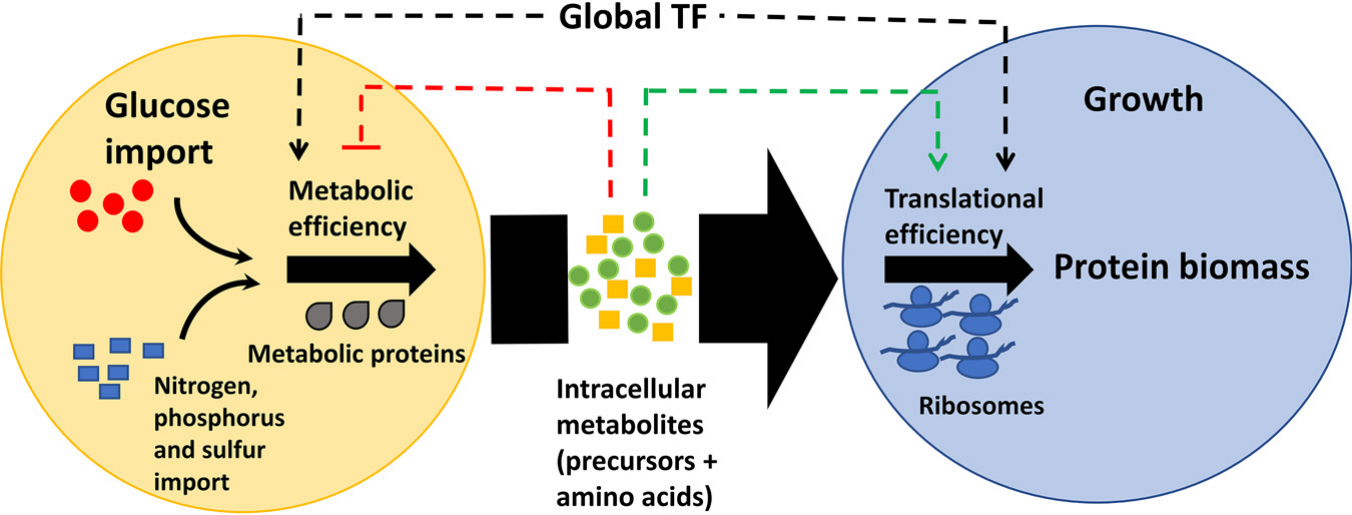
Model depicting global transcriptional factor (TF) control of the metabolic and translational proteome machinery. The yellow and blue circles represent processes related to glucose uptake and metabolism and to synthesis of biomass constituents toward growth, respectively. Carbon (represented as red circles) and nitrogen sources (ammonia, represented as blue squares), following their uptake, are processed into intracellular precursors (represented as yellow blocks) and amino acids (represented as green circles) depending on the metabolic efficiency of the organism. These intracellular amino acids are then converted into proteins by ribosomes depending on the translation efficiency of the organism. The stringent interplay between the metabolic and translation efficiency of the organism is coordinated by transcriptional regulators in conjunction with growth rate-dependent machinery. Any imbalance transduces the signal to metabolites that feedback regulate to restore control. In the absence of a regulator, the necessary genes utilizing metabolites and unnecessary genes diverting the metabolites from their cellular objectives cause a negative feedback effect, eventually causing a suboptimal growth rate. These metabolites can independently affect many cellular reactions, as evidenced by the known kinetics of metabolite regulation. Such detrimental changes are kept in check by global regulators complementing the linear association between growth and glucose import.

We elaborated on the role of these regulators emphasizing their direct and indirect control over key metabolic processes fundamental for balanced physiological growth of E. coli. For instance, the deletion of either FNR, ArcA, or IHF resulted in perturbations of key bottleneck steps of glycolysis, amino acid and nucleotide biosynthetic reactions, and derepression of the aerobic TCA cycle and alternate carbon metabolic proteins. Apart from carbon, we demonstrated that global regulators exhibit control over a nitrogen homeostasis and sensing mechanism as evident from the induction of high-affinity nitrogen transporters or scavengers and metabolite profiles as well as the altered ammonia uptake. Using the growth law theory (4, 7, 9, 11, 12, 28) and proteome-based ME model (36, 50, 51), we quantitatively accounted for this reduction in metabolic proteins and an increase in unnecessary or hedging proteins when an optimal WT strain is debilitated by global transcription regulator deletion. Owing to the huge energetic cost associated with ribosomal synthesis itself, demand for increasing translation to ameliorate the synthesis of necessary proteins further constrains the proteome share for metabolic proteins. We illustrate that disruption of this stringent trade-off between ribosomes and metabolic protein investments involved a lower translational and higher metabolic efficiency, thereby restraining growth (Fig. 7).

Transcriptionally regulating the first step or final committed step suffices to control the end product concentrations, be it the amino acid biosynthetic pathway or crucial reactions in central carbon metabolism (34, 57). The changes in the phenomenological parameters were manifested as accumulation of proteinogenic amino acids (glutamate, aspartate, and their derivatives) and precursors (PEP, pyruvate, αKG, and OAA), as evident from specific metabolic signatures in each of the regulator mutants. We suggest these amino acid accumulations are a consequence of not being efficiently utilized for protein biomass, as evident from the changes in efficiencies and increase in ribosomes, which may be suggestive of lower ppGpp levels. As we did not observe any changes in *lon* protease at the transcript level, the scenario wherein amino acid accumulations occur due to protein degradation promoted by higher ppGpp levels evident under starvation conditions seems less feasible (58). Intuitively, fueling products (particularly α-ketoacids, namely, pyruvate, αKG, and OAA) or building blocks (e.g., amino acids that on degradation generate α-ketoacids) have their feedback mechanisms on carbon import or amino acid biosynthesis that can independently affect the translational and metabolic proteome machinery (Fig. 7), limiting the potential of the system to attain a growth rate similar to that of the WT (12, 53–55, 57). Overall, in the absence of global regulators, we propose that the balance between growth rate and glucose uptake rate is attenuated, making the organism incapable to attain a fitter phenotype, unless forced upon a selection pressure (59, 60).

Though we explored the regulatory effects using quantitative measurements, we observed discrepancies in gene expression, metabolite profiles, and inferred protein activity of metabolic enzymes and coregulators, with their exact mechanism still unclear (53, 57). This could be addressed by accurate determinations of metabolites that had inconsistent responses (e.g., nucleotides) and by increasing the number of metabolites analyzed in each of the pathways to capture the overriding metabolite regulation. Furthermore, we note that variation might arise across different gene expression studies, but that would affect only the changes in efficiencies quantitatively. However, qualitatively, the trend of dysregulation would still hold true. Nevertheless, this work serves as a valuable template examining the coordination of genes and modulation of the activity of other transcription factors within the metabolic sector. Mechanistically, this delineated the complementary role of global transcriptional regulators with growth rate-dependent global machinery, predominant in exponentially growing cells. Such analyses can be extended to other global regulators such as cAMP receptor protein (CRP) (11, 59, 61), HNS (60), Lrp (62), and Fis (63), which tempts us to anticipate similar underlying mechanisms of regulation, presumably not confined only to glucose fermentative metabolism.

## MATERIALS AND METHODS

### Strain construction.

We constructed Δ*fnr*, Δ*arcA*, and Δ*ihf* (*ihfA-ihfB*) (see Table S2 in the supplemental material) knockouts in E. coli K-12 MG1655 (CGSC 6300), by λ Red-mediated recombination using the recombinase enzyme encoded plasmid pKD46, template plasmid pKD13 or pKD3 for the antibiotic selection cassette, and cured by pCP20 (64). The constructed strains were verified by PCR using the primers (Data Set S1, Primers_list sheet) covering on the region of interest as well as by Sanger sequencing. Glycerol stocks were made for each of the strains and stored at −80°C.

10.1128/mSystems.00001-21.9 TABLE S2 E. coli strains used in this study. Download Table S2, DOCX file, 0.01 MB. Copyright © 2021 Iyer et al. 2021 Iyer et al. https://creativecommons.org/licenses/by/4.0/ This content is distributed under the terms of the Creative Commons Attribution 4.0 International license.

### Physiological characterization in a bioreactor.

All characterizations were performed by growing cells in 400 ml M9 medium (6 g/liter anhydrous Na_2_HPO_4_, 3 g/liter KH_2_PO_4_, 1g/liter NH_4_Cl, 0.5 g/liter NaCl plus 2 mM MgSO_4_ plus 0.1 mM CaCl_2_) with 2 g/liter glucose in a 500-ml capacity bioreactor (Applikon). Briefly, cells from glycerol stocks were plated out on LB plus kanamycin (Kan; 50 μg/ml) agar plates, and a single colony was inoculated in LB medium. A fixed volume of 100 μl cells was used to inoculate 50 ml preculture M9 medium with 4 g/liter glucose, which was grown in a shake flask at 250 rpm in a 37°C (Eppendorf) incubator. The preculture cells, still in exponential phase, were centrifuged and washed with M9 medium (no carbon source) and inoculated in a bioreactor containing 400 ml M9 medium with 2 g/liter glucose such that the start optical density (OD) of all the cultures was ∼0.07. The temperature of the bioreactor was maintained at 37°C, the stirrer speed was 150 rpm, and the pH of the medium was maintained at pH 7.2 using 1 M NaOH. The pH was continuously monitored using a pH probe. Dissolved oxygen (DO) levels were monitored continuously using a polarographic dissolved oxygen probe and maintained at zero by constantly sparging nitrogen. The growth rate was measured from the slope of the linear regression line fit to the natural logarithm of the optical density values at 600-nm wavelength (OD_600_) versus time plot, ln(OD_600_) versus time (in hours), during the exponential growth phase. The dry cell weight was experimentally determined for each strain across the exponential phase such that an OD_600_ of 1.0 corresponds to 0.44 g (dry cell weight)·liter^−1^. Three biological replicates (*n* = 3) were considered for all phenotypic characterizations. To determine the rate (65) of glucose uptake as well as the rate of secretion of mixed acid fermentation metabolites (acetate, lactate, pyruvate, succinate, formate, and ethanol), samples were collected throughout the exponential phase which were then centrifuged. The supernatants were used to determine the concentrations using a high-pressure liquid chromatograph (HPLC) (Agilent 1200 series) equipped with a Bio-Rad Aminex HPX-87H ion exclusion column, with 5 mM H_2_SO_4_ as the mobile phase. The column temperature was maintained at 50°C and the flow rate at 0.6 ml/min. The supernatants were also used to determine the ammonia uptake rate using an enzyme-based assay (Sigma number AA0100). Given that pyruvate, succinate, and lactate are also secreted by the bacterial culture in substantial amounts, these metabolites were confined to exocellular metabolite measurements. Yields of biomass and mixed-acid fermentation products were calculated by normalizing their rates with glucose uptake rates (g/g glucose).

### RNA extraction and enrichment of mRNA.

The RNA extraction from two biological replicates of each strain was performed (*n* = 2). Briefly, the cells were grown anaerobically until the mid-exponential phase, after which 50 ml (OD of 0.35 to 0.4) cells were harvested by centrifugation. The TRIzol-chloroform method was used to extract total RNA (60, 66) as detailed below. DNase treatment was performed to remove any genomic DNA contamination. After DNase treatment, an initial enrichment of total RNA using a MegaClear kit (Ambion) was performed as per the manufacturer’s instructions. Enrichment of mRNA was performed using the MICROBExpress kit according to the manufacturer’s protocol, and the quality and integrity were checked using a Bioanalyzer. Paired-end strand-specific libraries for RNA sequencing were prepared using NEBNext Ultra directional RNA library kit, and the sequencing was carried out with HiSeq 4000 rapid run mode using 2 × 150 bp format at Genotypic Technologies, Bangalore, India.

### Transcriptome data analysis.

Raw read filtering, trimming, mapping, and alignment were performed as reported previously (59), taking into consideration the strand-specific paired-end reads. The error tolerance for filtering low-quality reads and adapter sequences was set to 20%, and trimmed reads with <50 bases were excluded. The annotations for 4,466 genes excluding the rRNA, tRNA, and small RNA (sRNA) genes were extracted from the EcoCyc database (version 21.5) (67). Raw counts from EdgeR (68) were then used for the analysis of differential gene expression, after filtering based on genes with reads of <0.5 counts per million (cpm). Differentially expressed genes (DEGs) showing ≥2-fold change in expression and adjusted *P* values of <0.05 (Benjamini-Hochberg) were used for all further analyses.

Enrichment of upregulated and downregulated DEGs was performed separately for regulators FNR, ArcA, and IHF using information available in EcoCyc (67), and statistical significance was performed using Fisher’s exact test. Only enrichments with a *P* value of <0.01 were considered for further analysis. For sigma factor enrichment analysis, the upregulated and downregulated DEGs were separately enriched based on sigma factor targets using data available in EcoCyc and RegulonDB (version 10.6.3) (69). The upregulated and downregulated genes under each regulator were then validated using a hypergeometric test in R (*P* value < 0.01). Only those sigma factors which regulated at least 5 genes from either the upregulated or downregulated DEGs in each of the mutants were retained for this overrepresentation analysis.

The upregulated and downregulated DEGs were separately enriched for metabolic pathways using KEGG pathway classification (70) as defined in Proteomaps (www.proteomaps.net), and the mapped genes were represented as Voronoi tree maps (version 2.0) (71). A hypergeometric test with *P* value correction using the Benjamini-Hochberg procedure was applied to determine the significance of the upregulated and downregulated genes within each pathway in R (R Core Team, version 2019). For each upregulated and downregulated pathway, we arbitrarily choose at least 10 DEGs to be considered for significance analysis. DEGs not having any assigned “Accession ID” (EcoCyc version 21.5) such as phantom genes were excluded from the analyses. The global and pathway-specific local regulators of E. coli K-12 and their cognate gene sets were downloaded from RegulonDB (version 10.6.3). The KEGG enriched up- and downregulated DEGs were separately analyzed for enrichment of global and local regulators. The global and local regulators found to be significant by Fischer’s exact test (*P* < 0.01) were retained for further analysis (Data Set S1, Reg enrichment sheets for *fnr*, *arcA*, and *ihf*). An arbitrary cutoff of at least 5 genes was chosen to be considered for enrichment analysis.

The ppGpp enrichment analysis was performed on the complete list of upregulated and downregulated DEGs separately using data available in EcoCyc, and statistical significance was assessed using Fisher’s exact test. Only enrichments with a *P* value of <0.01 were considered for further analysis.

### Quantitative RT-PCR validation for RNA-seq.

Quantitative reverse transcription-PCR (qRT-PCR) was performed with the Agilent AriaMx machine using the PowerUp SYBR green PCR master mix to validate the RNA-seq data. *rpoB* was used as an internal control to normalize the qRT-PCR data. DNase-treated total RNA to cDNA conversion was performed using Superscript IV reverse transcriptase (Invitrogen) according to the manufacturer’s instructions. All experiments were performed in biological duplicates and technical triplicates (*n* = 6). The comparative threshold cycle (2^−ΔΔ^*^CT^*) method described previously (72) was used to quantify expression fold changes. We observed a strong correlation (>0.93) between qRT-PCR and RNA-seq data (Data Set S1, qRT-PCR_validation sheet).

### Metabolomics.

Metabolite samples were harvested from anaerobically growing cells in the mid-exponential phase. Three biological and two technical replicates (*n* = 6) were harvested as reported previously (59) with minor changes specific to anaerobic conditions. A fast-cooling method was used to quench the harvested cells as reported previously (73, 74). Briefly, ∼15 ml culture (cells at an OD of >∼5) was rapidly poured into 5 ml chilled M9 medium (without glucose) in a precooled 50-ml falcon tube. To rapidly bring the temperature of the sample tube down to 0°C, the tube was dipped in liquid nitrogen for 10 s with vigorous agitation with the help of a digital thermometer, to prevent ice crystal formation. Samples were then immediately centrifuged at 0°C at 7,800 rpm for 7 min. The supernatant was discarded, and the pellet was snap-frozen in liquid nitrogen and stored at −80°C until metabolite extraction was performed.

For metabolite extraction, 7:3:5 (vol/vol/vol) methanol-chloroform-ammonium hydroxide (2% [wt/vol]) was used as described previously (59, 74). ^13^C-labeled E. coli extracts were used as internal standards that were generated separately under aerobic flask conditions using the wild-type strain. The labeled extracts were used in the quantification of key metabolite pool sizes using an isotope-based dilution method (75). All the extracted samples were spiked with a fixed volume of internal standard taken from the same batch at an early stage of extraction. The volume of the pooled internal standard added to the samples was accepted only if the external ^12^C peak height (concentration similar to samples) and internal ^13^C standard peak height differed <5-fold (54). The liquid chromatography-tandem mass spectrometry (LC-MS/MS) settings (59) and chromatographic conditions (59, 76) were maintained as reported previously. Cleaning and maintenance of the LC-MS systems were performed (77) before the actual setup. The electrospray ionization (ESI) was operated in positive [M+H]^+^ and negative [M−H]^−^ mode separately. MS1 parent ion was used for quantification purposes. The MS2 setting was used for secondary validation of metabolites, including the ^12^C chemical standards.

### Metabolomics data analysis.

The raw files generated from the machine were processed using the software package Xcalibur 4.3 (Thermo Fisher Scientific) Quan Browser, as has been shown in a previous study (59). Quantitative analysis was performed wherein the peak heights of precursor ions with a signal/noise (S/N) ratio of more than 3 and less than 5 ppm error were considered. The metabolite height ratios were obtained after normalizing the peak heights of the samples to the peak height of the internal standards. To identify the concentrations, serial dilutions of ^12^C chemical standards (mix of 40 metabolites) supplemented with the fixed volume (as in samples) from the same batch of ^13^C-labeled internal standards were used to generate a calibration curve in the range of 0.781 μM to 50 μM. These standards were run in biological duplicates (*n* = 2) for the above-mentioned concentration range in the positive and negative modes separately. All metabolite concentrations were within the calibration curve range, and those which did not fall in this range were individually checked for that particular concentration to assess whether they lie within the limit of quantification (LOQ) (S/N = 10).

MetaboAnalyst (78) was used for identifying statistically significant metabolites. Biomass normalized concentrations on metabolites were g-log transformed before analysis. Missing value imputation was performed using the SVD impute function in MetaboAnalyst. The absolute concentration of metabolites is expressed as micromoles per gram dry cell weight. Only those metabolites with a false-discovery rate (FDR) value of <0.05 (two-tailed unpaired Student’s *t* test) were considered for further analysis.

We sought to identify the specific pattern of precursor or amino acid correlations with the glucose uptake and growth rate across the WT and mutant conditions given that the physiological state of the system was perturbed due to a regulator deletion. Towards this, we performed pairwise Pearson correlation analysis (Pearson product-moment correlation) with statistical significance (*P* < 0.05) between metabolites and phenomic features such as glucose uptake, growth rate, etc., in each of the mutants compared to that of the WT in R. For this analysis, only features/metabolites found to be statistically significant (FDR < 0.05 from Student’s *t* test) in each mutant compared to that in the WT were considered for correlation analysis. The concentrations (*n* = 6) of technical and biological replicates of metabolites were clubbed (*n* = 3) to make them comparable with phenomic features (*n* = 3). These concentrations or rates were log_2_ normalized before assessing the correlation in R (R Core Team, version 2019).

### ME model simulations.

We assessed the utilized ME and nonutilized ME protein-coding genes, as reported previously (59), using an E. coli ME model (50, 51). The simulation involved constraining the glucose uptake rates in the range from zero to unbounded glucose (36, 59, 79), with an additional constraint on oxygen that was set to zero and the maximization growth rate as an objective function. Genes predicted to have a reasonable protein translation flux (≥10^−15 ^mmol/g [dry cell weight]/h) in any of the simulations were classified as “utilized ME;” genes within the scope of the ME model that showed no expression or very low expression in any of the simulations (protein translation flux < 10^−15 ^mmol/g [dry cell weight]/h) were classified as “nonutilized ME” (Data Set S1, ME_nonME_genes_anaerobic sheet). It should be noted that from the predicted gene list, only the genes encoding metabolic proteins specific to glucose metabolism as a function of growth rate were considered and annotated as “M” sector and unnecessary/unused “U” sector (ribosome-affiliated proteins were not considered). Two assumptions were considered in this analysis: (i) DEGs mapped to the utilized ME and nonutilized ME genes correspond to the metabolic “M” sector and unnecessary/unused “U” sector genes, respectively, and (ii) the increase in these protein-coding transcriptome fractions corresponds to an increase in proteome fractions. DEGs utilized in the simulation correspond to genes that were differentially expressed (Benjamini-Hochberg adjusted *P* values < 0.05, adjusted fold change [aFC] ≥ 2) in at least one condition (Δ*fnr* versus WT, Δ*arcA* versus WT, and Δ*ihf* versus WT). We mapped the DEGs in each of the mutants compared to WT to the ME model-predicted protein-coding genes to collectively account for M sector and U sector genes. This mapping was performed utilizing the combined list of DEGs from all the mutants to make it comparable across the WT and all mutant strains. Additionally, DEGs (excluding transcriptional regulator genes) outside the scope of the ME model were further enriched using KEGG pathway classification as well as based on manual annotation using EcoCyc, which were then added to M sector and U sector gene lists. Raw counts (genes with <0.5 cpm reads were not considered) for all the 4,466 genes in WT and the mutants were used for calculation of transcript per million (TPM) (80). Furthermore, the genes with lengths less than the mean fragment lengths of paired-end reads were filtered out of the TPM analysis. The mean fragment lengths were computed using Picard tools (http://broadinstitute.github.io/picard/). TPMs were assigned to all the M sector and U sector genes annotated to DEGs. The transcriptome fraction was calculated using the product of TPM and gene length divided by the sum product of these calculated over the sector-specific genes (Data Set S1, Transcriptome fractions sheet). Finally, we summed the transcriptome fractions of all M sector and U sector genes independently (36). These fractions were scaled to percentages and depicted in Fig. 6A. The U/M ratio was calculated by dividing these protein-coding transcriptome fractions of the U sector and M sector for each of the strains.

### Total RNA estimation.

Total RNA extracted from the cells using the TRIzol-chloroform method as per the manufacturer’s instructions. Briefly, 50 ml cells were harvested from the bioreactor at and OD of around 0.35 to 0.4. Cells were pelleted by centrifugation at 4°C. The pellets were stored at −20°C until utilized for further processing steps. The pellet was snap-frozen in liquid nitrogen followed by the addition of 300 μl TRIzol. The pellet was homogenized using a hand-held pestle for not more than 2 min. To this homogenized pellet, 700 μl TRIzol was added, which was then vortexed and kept on ice for 15 min. Further extraction was performed using chloroform (300 μl), and the sample was centrifuged to separate the protein, DNA, and RNA layers. The RNA layer was then precipitated using isopropanol as per the instructions provided for the TRIzol method. Next, RNA was centrifuged at 4°C, and the RNA pellet was washed with 70% (vol/vol) ethanol at room temperature. The pellet was dried by inverting the tubes, making sure there were no traces of ethanol, and to the dried pellet, 30 to 40 μl DNase-RNase free water was added. This reconstituted sample was incubated in a dry bath at 55°C for 10 min, brought to room temperature, and then stored as aliquots at −80°C until use. The total RNA was checked for its purity and integrity by running on an agarose-formaldehyde gel and Bioanalyzer, and its concentration was estimated using a Nanodrop. The obtained RNA concentration (μg/ml) was normalized to its respective OD at 600 nm and gram (dry cell weight) conversion factor (0.44 mg/ml) to derive the final concentration (μg/mg [dry cell weight]).

### Total protein estimation.

The total protein quantification was determined using 1.8 ml of culture at an OD of around 0.35 to 0.4 and was based on the Biuret method described previously (11). Briefly, the cells were immediately centrifuged at 4°C and the supernatant was discarded. The pellet was washed once with autoclaved and chilled Milli-Q water with another round of centrifugation. The pellet was dissolved with 200 μl of autoclaved and chilled Milli-Q water followed by snap-freezing in liquid nitrogen and storage at −20°C until use. The pellets were thawed at room temperature. Once thawed, 100 μl of 3 M NaOH was added to the suspension and mixed before incubation at 100°C for 5 min to liberate the proteins by cell lysis. After cooling the lysate to room temperature, 100 μl of 1.6% (wt/vol) CuSO_4_ was added with vigorous mixing for 5 min to initiate the Biuret reaction. After centrifugation, the colored solution was diluted to 1:1 with autoclaved and chilled Milli-Q water to measure absorbance at 555 nm in a MultiScan GO (Thermo Scientific) spectrophotometer using a cuvette. Milli-Q water treated with the NaOH and CuSO_4_ step was used as the blank. The bovine serum albumin (BSA) standard was also subjected to the same conditions as the sample to analyze the concentration of proteins. The slope obtained from the standard curve was used for the calculation of protein concentrations. The obtained protein concentration (μg/ml) was normalized to its respective OD at 600 nm and gram (dry cell weight) conversion factor (0.44 mg/ml) to derive the final concentration (μg/mg [dry cell weight]).

### Data availability.

The RNA sequencing data and the processed files from this study are available at NCBI Geo under accession number GSE153906. The metabolomics data presented in this study are available at the NIH Common Fund’s National Metabolomics Data Repository (NMDR) website, the Metabolomics Workbench, where it has been assigned project identifier (ID) PR000975. The data can be accessed directly via https://www.metabolomicsworkbench.org/data/DRCCMetadata.php?Mode=Project&ProjectID=PR000975.
