# Supplementary figures and images for: Global Transcriptional Regulators Fine-Tune the Translational and Metabolic Efficiency for Optimal Growth of Escherichia coli
Source: mSystems. 2021 Mar 30;6(2):e00001-21. doi: 10.1128/mSystems.00001-21 (PMC8546960; doi:10.1128/mSystems.00001-21)

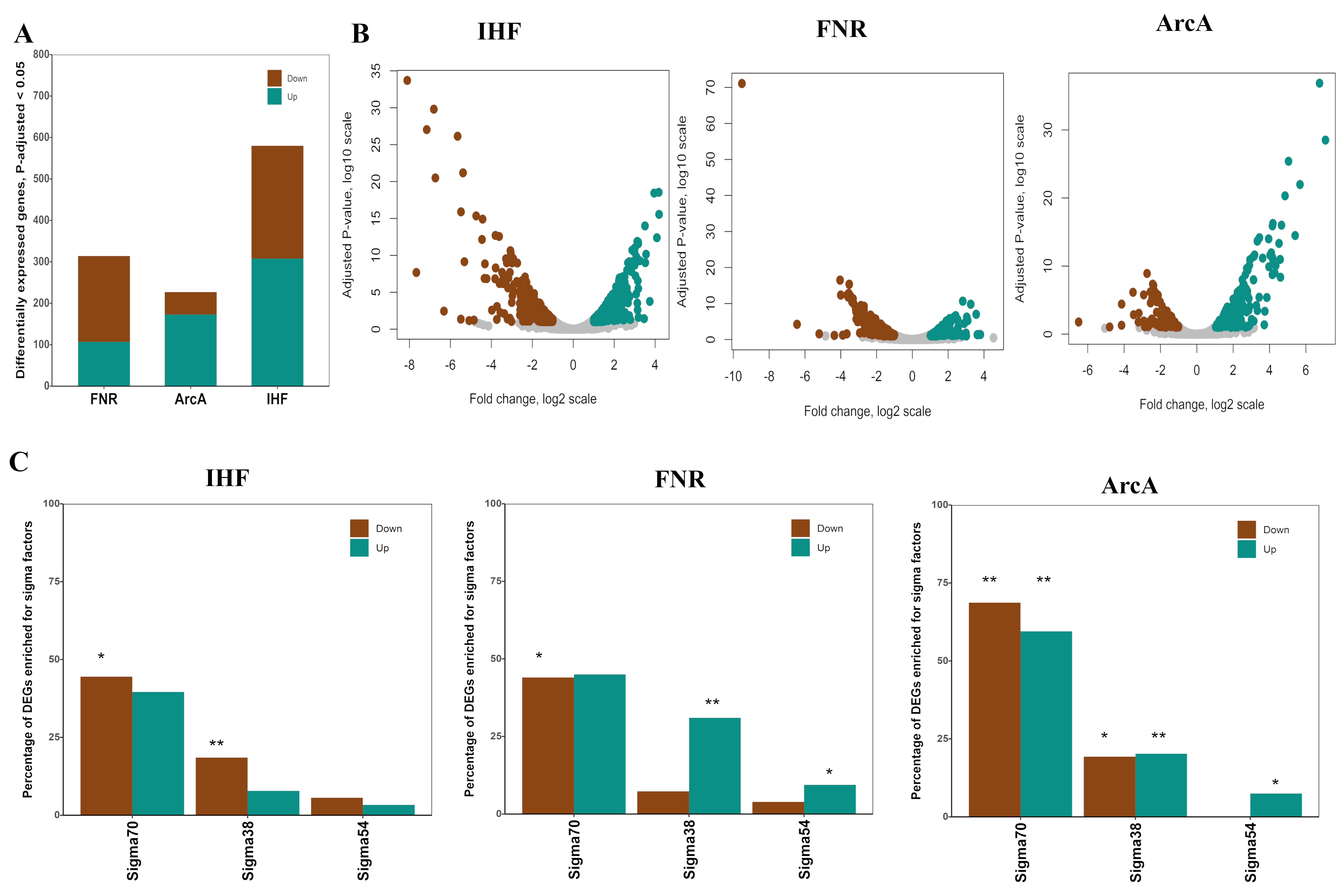

Supplement: FIG S1 [file msystems.00001-21-sf001.tif]

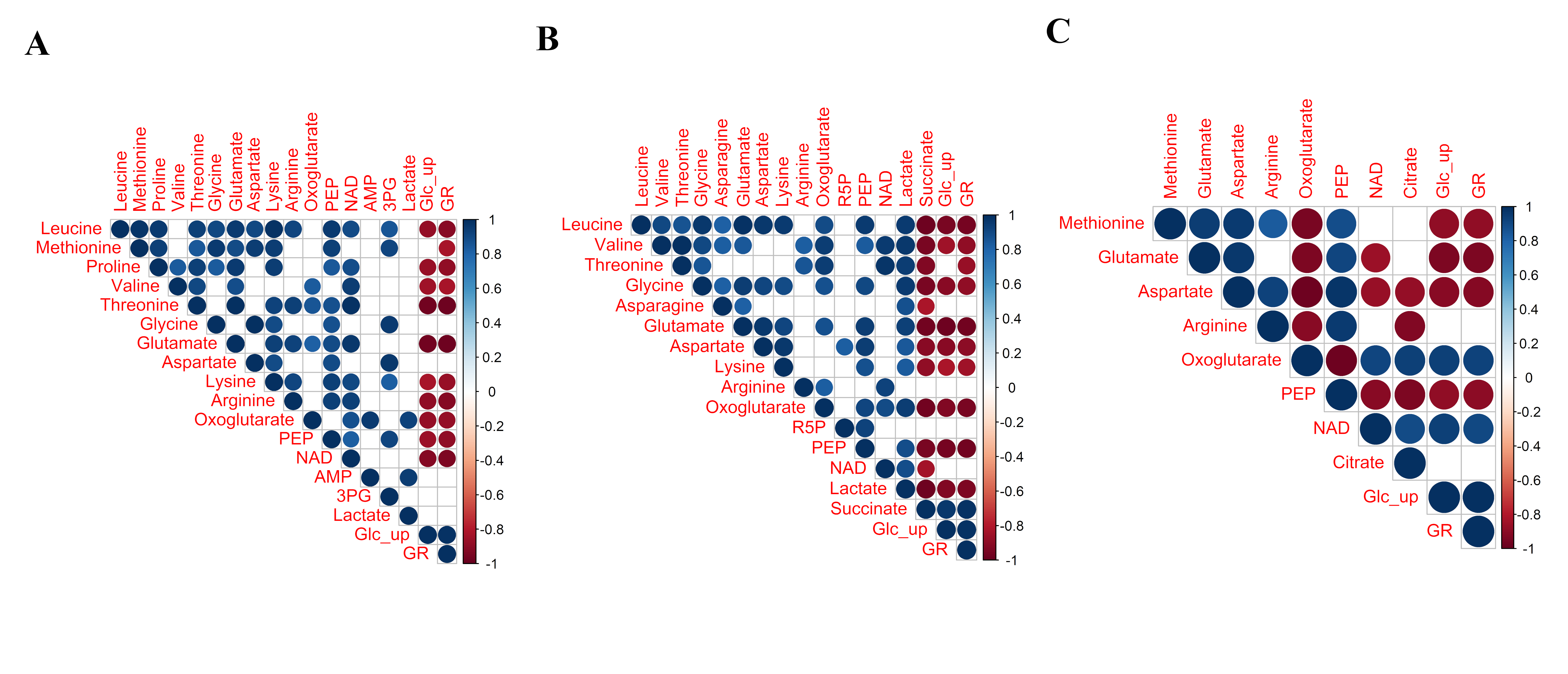

Supplement: FIG S6 [file msystems.00001-21-sf006.tif]
