# Supplementary material for: Global Transcriptional Regulators Fine-Tune the Translational and Metabolic Efficiency for Optimal Growth of Escherichia coli
Source: mSystems. 2021 Mar 30;6(2):e00001-21. doi: 10.1128/mSystems.00001-21 (PMC8546960; doi:10.1128/mSystems.00001-21)
Supplement: TABLE S1 [file msystems.00001-21-st001.docx]

| Strains | Growth rate  (h^-1^) | Glucose uptake rate  (mmol/gDCW/h) | Yield of Ethanol  (g/g glucose) | Yield of Formate  (g/g glucose) | Yield of Acetate  (g/g glucose) | Yield of Lactate  (g/g glucose) | Yield of Succinate  (g/g glucose) | Yield of Pyruvate  (g/g glucose) | Yield of Biomass  (g/g glucose) | Ammonia uptake rate  (mmol/gDCW/h) |
| --- | --- | --- | --- | --- | --- | --- | --- | --- | --- | --- |
| WT | 0.38 ± 0.007 | 16.63 ± 0.5 | 0.18 ± 0.01 | 0.35 ± 0.01 | 0.21 ± 0.01 | 0.02 ± 0 | 0.09 ± 0.01 | 0.02 ± 0 | 0.13 ± 0 | 6.07 ± 0.5 |
| Δ*ihf* | 0.29 ± 0.007 | 13.26 ± 0.23 | 0.19 ± 0 | 0.36 ± 0.01 | 0.23 ± 0 | 0.04 ± 0.017 | 0.07 ± 0.03 | 0.003 ± 0 | 0.12 ± 0 | 4.76 ± 0.63 |
| Δ*fnr* | 0.29 ± 0.008 | 12.12 ± 0.62 | 0.18 ± 0.01 | 0.39 ± 0.02 | 0.23 ± 0 | 0.04 ± 0 | 0.05 ± 0 | 0.002 ± 0 | 0.13 ± 0.01 | 4.4 ± 0.53 |
| Δ*arcA* | 0.32 ± 0.015 | 13.99 ± 0.44 | 0.18 ± 0 | 0.33 ± 0.02 | 0.21 ± 0.01 | 0.01 ± 0 | 0.12 ± 0.01 | 0.002 ± 0 | 0.13 ± 0 | 4.99 ± 0.21 |

**Table S1:** Physiological characterization of the strains in anaerobic fermentation of glucose. The measurements of growth rate, glucose and ammonia uptake rate, and yields of mixed acid fermentation products were obtained from three biological replicates (n = 3). Yields were calculated by normalizing secretion rates with its glucose uptake rate. The errors indicate standard deviations within the replicates.
