# Supplementary material for: Global Transcriptional Regulators Fine-Tune the Translational and Metabolic Efficiency for Optimal Growth of Escherichia coli
Source: mSystems. 2021 Mar 30;6(2):e00001-21. doi: 10.1128/mSystems.00001-21 (PMC8546960; doi:10.1128/mSystems.00001-21)
Supplement: TABLE S2 [file msystems.00001-21-st002.docx]

| **Strain Name** | **Genotype** | **Source** |
| --- | --- | --- |
| *E. coli* K12 MG1655 WT | F-, *λ-*, *ilvG- rfb-50* *rph-1* | Keio collection *CGSC #6300* |
| *E. coli* K12 MG1655 Δ*arcA* | F-, *λ-*, *ilvG- rfb-50* *rph-1,* Δ*arcA::kan* | This study |
| *E. coli* K12 MG1655 Δ*fnr* | F-, *λ-, ilvG- rfb-50* *rph-1*, Δ*fnr::kan* | This study |
| *E. coli* K12 MG1655 Δ*ihf* | F-, *λ-, ilvG- rfb-50* *rph-1*, Δ*ihfA::FRT* Δ*ihfB::kan* | This study |

**Table S2.** The E. coli strains used in this study.
